# Global temporal patterns of pancreatic cancer and association with socioeconomic development

**DOI:** 10.1038/s41598-017-02997-2

**Published:** 2017-06-09

**Authors:** Martin C. S. Wong, Johnny Y. Jiang, Miaoyin Liang, Yuan Fang, Ming Sze Yeung, Joseph J. Y. Sung

**Affiliations:** 1 School of Public Health and Primary Care, Faculty of Medicine, Chinese University of Hong Kong. Prince of Wales Hospital, Shatin, New Territories, Hong Kong, China; 2 Institute of Digestive Disease, Faculty of Medicine, Chinese University of Hong Kong, Hong Kong, China; 3 Chinese Academy of Medical Sciences and Peking Union Medical College, Hong Kong, China; 4 Department of Medicine and Therapeutics, Faculty of Medicine, Chinese University of Hong Kong, Hong Kong, China; 5 State Key Laboratory of Digestive Disease, Faculty of Medicine, Chinese University of Hong Kong, Hong Kong, China

## Abstract

Pancreatic cancer induces a substantial global burden. We examined its global incidence/mortality rates and their correlation with socioeconomic development (Human Development Index [HDI] and Gross Domestic Product [GDP] in 2000 as proxy measures). Data on age-standardized incidence/mortality rates in 2012 were retrieved from the GLOBOCAN database. Temporal patterns in 1998–2007 were assessed for 39 countries according to gender. The Average Annual Percent Change (AAPC) of the incidence/mortality trends was evaluated using joinpoint regression analysis. The age-standardized incidence ranged between 0.8–8.9/100,000. When compared among countries, Brazil (AAPC = 10.4, 95%C.I. = 0.8,21) and France (AAPC = 4.7, 95%C.I. = 3.6,5.9) reported the highest incidence rise in men. The greatest increase in women was reported in Thailand (AAPC = 7, 95%C.I. = 2.1,12.1) and Ecuador (AAPC = 4.3, 95%C.I. = 1.3,7.3). For mortality, the Philippines (APCC = 4.3, 95%C.I. = 2,6.6) and Croatia (AAPC = 2, 95% C.I. = 0,3.9) reported the biggest increase among men. The Philippines (AAPC = 5.8, 95% C.I. 4.5,7.2) and Slovakia (AAPC = 3.1, 95% C.I. 0.9,5.3) showed the most prominent rise among women. Its incidence was positively correlated with HDI (men: r = 0.66; women: r = 0.70) and GDP (men: r = 0.29; women: r = 0.28, all p < 0.05), and similarly for mortality (men: r = 0.67; women: r = 0.72 [HDI]; men: r = 0.23; women: r = 0.28 [GDP]). In summary, the incidence and mortality of pancreatic cancer were rising in many countries, requiring regular surveillance.

## Introduction

Worldwide, pancreatic cancer is the twelfth most common malignancy and the seventh leading cause of cancer mortality, with more than 330,000 deaths in 2012^1, 2^. In 2008, the global disease burden attributable to this cancer has been estimated at around 126 per 100,000 age- and disability-adjusted life years (DALY) with a substantial number of years of life lost^3^. The remote location of the pancreas; the lack of appropriate screening tests or diagnostic markers; the aggressiveness of pancreatic adenocarcinoma; its poor response to chemotherapy or radiotherapy; and the difficulties to establish a tissue diagnosis led to the low success rates of its treatment^4^. Owing to its extremely aggressive nature and poor survival rate^4^, it remains an important public health issue worldwide.

Most pancreatic cancers were diagnosed in northern and more developed countries^2^. The vast majority of pancreatic cancers are adenocarcinoma, with slower-growing endocrine tumors accounting for other pancreatic cancer subtypes^5^. The recognized risk factors for pancreatic cancer include cigarette smoking^6^ and use of smokeless tobacco^7^. Some possible risk factors include those that are related to lifestyle factors^8–16^.

There is a strong prospect to monitor the incidence and mortality of pancreatic cancer by disease surveillance. Some projection studies have estimated that pancreatic cancer would escalate from the fourth to the second leading cause of cancer deaths in the United States by 2020^17^. Hence, a comprehensive evaluation of its epidemiology with respect to its global pattern and trends is crucial to inform resource planning for future healthcare service provision.

Previous studies and review articles describing the international trends of pancreatic cancer were based on figures from registries in 2002 to 2009^4, 5, 18^. They did not take into account the socioeconomic development of each country when comparisons were made, and depended on comparison among countries in a single calendar year^4, 10, 11^. At a global level, it is still uncertain whether differential effect of socioeconomic status on the risk of developing pancreatic cancer exists, which is as an important knowledge gap. This study aims to delineate the patterns and temporal trends of pancreatic cancer in 184 and 39 countries, respectively, based on data from high quality cancer registries. We also tested the *a priori* hypothesis that the incidence and mortality of pancreatic cancer were associated with differences in socioeconomic development and productivity across different countries.

## Methods

### Data Source

The incidence and mortality estimates for pancreatic cancer (ICD-10 C22) were retrieved from the GLOBOCAN database in 2012^2^. We made reference to recent analyses of epidemiological data on colorectal^19^, liver^20^, and prostate cancer^21^, and used similar methodology to evaluate the patterns and trends of pancreatic cancer. We obtained data on the Human Development Index (HDI) and Gross Domestic Product (GPD) for each country in 2000 from the United Nations Human Development Report^21^. HDI is a composite index of life expectancy, education period, and income per capita indicators, and is perceived as “an index of potential human development”^22^. Whilst the GDP per capita measures the size of an economy by summing up the value of goods and services produced within the country in a specific period of time, the HDI takes into account GDP and also other factors that measure other aspects of human development, including knowledge, longevity and decent standard of living. To examine time trends, information was retrieved from different sources where at least 10 consecutive years of data could be obtained. We included data for countries where both incidence and mortality figures were available. For incidence figures, we extracted high-quality national population-based cancer registries from the *Cancer Incidence in Five Continents* (CI5) series Volumes I-X^23^. To include incidence data for more recent years, we also utilized publicly available information from the U.S.^24^, European countries^25–27^, Australia^28^, and New Zealand^29^. The incidence data for pancreatic cancer were allocated into different categories according to the International Classification of Diseases 10^th^ revision (ICD-10 C22), whereas mortality data were categorized based on the ICD 9^th^ (155) up to 1991 and 10^th^ version (C22) thereafter^30^. When there are duplicates of incidence or mortality figures obtained from the CI5 and the regional registries, data from the former source were used in the analysis. For instance, we used the data from the national database at Australian Institute of Health and Welfare where the incidence and mortality figures were more comprehensive. In total, we examined the temporal trends of incidence and mortality in 39 countries. This study has been approved by the Survey and Behavioural Research Ethics Committee of the Chinese University of Hong Kong. As this study used routinely collected anonymised electronic data consent was not required.

For mortality data, we made reference to the WHO mortality data series where data quality attained criteria of medium level or above^31^, which resulted in data with extensive coverage as well as high accuracy and completeness. Death certificates acted as the primary data source, and were compiled by the International Agency for Research on Cancer (IARC) as part of the WHO mortality database. We adopted age-standardized rates (ASR) using the world standard population^32^. Similar to the IARC, we defined more developed countries as all regions of Europe plus Northern America, Australia/New Zealand and Japan, and less developed regions as all regions of Africa, Asia (excluding Japan), Latin America and the Caribbean, Melanesia, Micronesia and Polynesia^2^.

### Statistical Analysis

We employed joinpoint regression analysis to examine the incidence and mortality trends^33^, using the joinpoint statistical software version 3.4. This technique fits a series of joined straight lines to the trend of ASR^33^. Logarithmic transformation of the rates was performed with computation of the standard errors based on binomial approximation. We specified a maximum number of three joinpoints as analysis options, as adopted by Arnold *et al*. and our previous study^20, 21^. To determine the direction and magnitude of the recent trends, the average annual percentage change (AAPC) and the respective 95% confidence intervals were evaluated where data were available in the most recent 10 years in the period 1998 to 2012. The AAPC was calculated as a geometrically weighted average of the various APCs from the joinpoint regression analysis, with weights being equivalent to the length of each segment during the specified time interval^34^. The statistical significance of AAPC was ascertained comparing its magnitude with zero, and all insignificant AAPCs were regarded as having “stable trend”.

The ASRs were plotted against the HDI and GDP per capita, respectively. The HDI was divided into four distinct categories, including low (≤0.534), medium (0.534 < HDI ≤ 0.710), high (0.710 < HDI ≤ 0.796) and very high (HDL > 0.796) based on the United Nations Human Development Report in 2012^22^. Simple linear regression was conducted and correlation coefficients were estimated to examine their associations and the goodness-of-fit. All p values < 0.05 were regarded as statistically significant.

## Results

### Incidence and mortality of pancreatic cancer in 2012

A total of 337,872 new cases of pancreatic cancer and 330,391 related deaths were reported in 2012. Approximately 55% of the total incidence and 56% of all mortality occurred in more developed regions. The ratio between the ASR of incidence and mortality was similar between more developed and less developed countries (1.02 vs. 1.03) in both male and female subjects. Among all continents, the Oceania including Australia and New Zealand had the highest incidence to mortality ratio (1.11). The age-standardized incidence rates of pancreatic cancer ranged from 0.8 to 8.9 per 100,000 in 2012. Among men, the highest were found in Central and Eastern Europe (ASR 8.9 per 100,000), North America (8.5), Western Europe (8.3) and Southern Europe (7.6), and the lowest were reported in South-Central Asia (1.3), Middle Africa (1.4) and Eastern Africa (1.5) (Table 1). Among women, the highest were found in North America (ASR 6.4 per 100,000), Western Europe (6.3), Northern Europe (5.9) and Australia/New Zealand (5.4), whereas the lowest were reported in Middle Africa (0.8), South-Central Asia (1.0), Melanesia (1.1) and Western Africa (1.3) (Table 2).

**Table 1:** The estimated incidence and mortality of pancreatic cancer according to world area, 2012, males.

World regions	Population size (million)	Incidence (I)		Mortality (M)		I:M ratio
		n	ASR	n	ASR	
**Africa**	1072.3	6625	2.3	6424	2.3	1.03
Eastern Africa	342.3	1228	1.5	1204	1.5	1.02
Middle Africa	134.1	417	1.4	401	1.3	1.04
Northern Africa	213.0	2499	3.3	2430	3.2	1.03
Southern Africa	58.8	958	5.3	924	5.2	1.04
Western Africa	324.1	1523	2.0	1465	1.9	1.04
**Asia**	4260.0	80704	3.8	76698	3.6	1.05
Eastern Asia	1585.0	60979	5.5	58037	5.2	1.05
South-Eastern Asia	607.9	6413	2.5	6207	2.5	1.03
South-Central Asia	1822.9	9299	1.3	8560	1.2	1.09
Western Asia	243.8	4013	4.7	3894	4.7	1.03
**America**	948.2	13067	4.6	13312	4.7	0.98
Caribbean	42.0	1002	4.2	989	4.1	1.01
Central America	160.2	2521	3.7	2413	3.6	1.04
South America	397.2	9544	5.0	9910	5.1	0.96
North America	348.8	23949	8.5	23165	8.0	1.03
**Europe**	740.1	51962	8.2	52631	8.2	0.99
Central and Eastern Europe	295.0	17774	8.9	18052	9.0	0.98
Northern Europe	100.6	6938	7.3	6967	7.1	1.00
Southern Europe	154.2	11547	7.6	11511	7.4	1.00
Western Europe	190.4	15703	8.3	16101	8.0	0.98
**Oceania**	37.0	1854	7.0	1597	5.9	1.16
Australia/New Zealand	26.4	1778	7.5	1520	6.3	1.17
Melanesia	10.2	59	2.4	56	2.3	1.05
Micronesia/Polynesia	0.4	17	3.1	21	3.9	0.81
More developed regions	1242.9	94702	8.6	93125	8.3	1.02
Less developed regions	5815.1	83459	3.3	80702	3.2	1.03
**World**	7058.0	178161	4.9	173827	4.7	1.02

ASR = Age standardized rate per 100,000. Source: GLOBOCAN 2012 [1]. Numbers are rounded to the nearest 10 or 100, and may not add up to the total. The population size of the world regions were retrieved from the Population Reference Bureau, Washington, DC. Available at: http://www.prb.org/Publications/Datasheets/2012/world-population-data-sheet/world-map.aspx#/table/population.

**Table 2:** The estimated incidence and mortality of pancreatic cancer according to world area, 2012, female.

World regions	Population size (million)	Incidence (I)		Mortality (M)		I:M ratio
		n	ASR	n	ASR	
**Africa**	1072.3	5476	1.7	5280	1.6	1.04
Eastern Africa	342.3	1602	1.7	1546	1.6	1.04
Middle Africa	134.1	278	0.8	266	0.7	1.05
Northern Africa	213.0	1535	1.8	1485	1.8	1.03
Southern Africa	58.8	911	3.5	883	3.4	1.03
Western Africa	324.1	1150	1.3	1100	1.2	1.05
**Asia**	4260.0	62659	2.6	60553	2.5	1.03
Eastern Asia	1585.0	46392	3.6	45080	3.4	1.03
South-Eastern Asia	607.9	5869	2.0	5651	1.9	1.04
South-Central Asia	1822.9	7511	1.0	6999	0.9	1.07
Western Asia	243.8	2887	3.1	2823	3.0	1.02
**America**	948.2	14656	4.2	14623	4.1	1.00
Caribbean	42.0	1012	3.6	1034	3.6	0.98
Central America	160.2	2906	3.7	2801	3.5	1.04
South America	397.2	10738	4.4	10788	4.4	1.00
North America	348.8	23422	6.4	22651	5.9	1.03
**Europe**	740.1	51883	5.5	51923	5.3	1.00
Central and Eastern Europe	295.0	16889	5.0	16965	4.9	1.00
Northern Europe	100.6	7305	5.9	7315	5.6	1.00
Southern Europe	154.2	11522	5.3	11236	4.9	1.03
Western Europe	190.4	16167	6.3	16407	5.8	0.99
**Oceania**	37.0	1615	5.0	1534	4.6	1.05
Australia/New Zealand	26.4	1572	5.4	1493	4.9	1.05
Melanesia	10.2	35	1.1	35	1.2	1.00
Micronesia/Polynesia	0.4	8	1.4	6	1.1	1.33
More developed regions	1242.9	92763	5.9	91304	5.5	1.02
Less developed regions	5815.1	66948	2.4	65260	2.3	1.03
**World**	7058.0	159711	3.6	156564	3.4	1.02

ASR = Age standardized rate per 100,000. Source: GLOBOCAN 2012 [1]. Numbers are rounded to the nearest 10 or 100, and may not add up to the total. The population size of the world regions were retrieved from the Population Reference Bureau, Washington, DC. Available at: http://www.prb.org/Publications/Datasheets/2012/world-population-data-sheet/world-map.aspx#/table/population.

Worldwide, the mortality rates of pancreatic cancer ranged from 0.7 to 9.0 per 100,000 in 2012. In men, the highest mortality rates were reported in Central and Eastern Europe (ASR 9.0 per 100,000), North America (8.0), Western Europe (8.0) and Southern Europe (7.4) (Table 1); whilst in women, the highest mortality was also reported in these four regions (ASR mortality = 4.9, 5.9, 5.8 and 4.9 per 100,000, respectively). The lowest mortality rates were found in South-Central Asia (1.2), Middle Africa (1.3) and Eastern Africa (1.5) in men. For women, Middle Africa (0.7), South-Central Asia (0.9), Micronesia/Polynesia (1.1) and Western Africa (1.2) reported the lowest mortality rates. Countries having the highest incidence to mortality ratios in men included Australia/New Zealand (1.17) and South-Central Asia (1.09), and the ratios were the highest for women in Micronesia/Polynesia (1.33) and South-Central Asia (1.07).

### The relationship between incidence/mortality of pancreatic cancer and socioeconomic development

**Figure 1:**
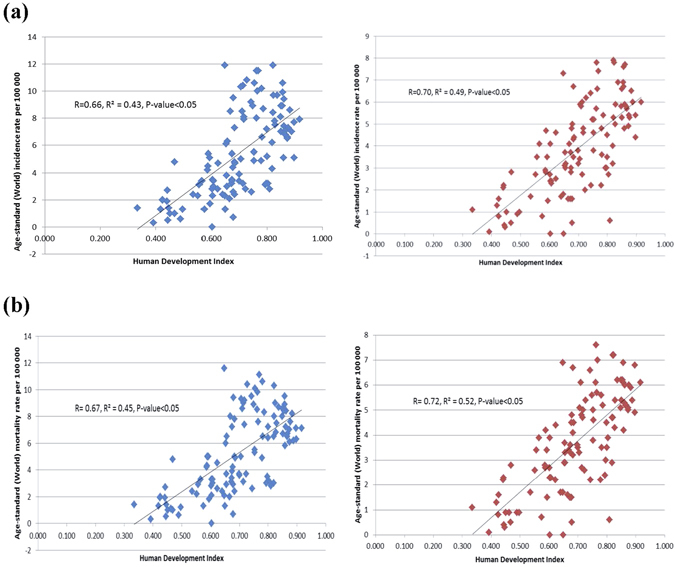
(**a**) Correlation between age-standardised incidence of pancreatic cancer in 2012 and Human Development Index (HDI) in 2000 in men (left) and women (right); (**b**). Correlation between age-standardised incidence of pancreatic cancer in 2012 and Gross Domestic Product (GDP) per capita in 2000 in men (left) and women (right).

**Figure 2:**
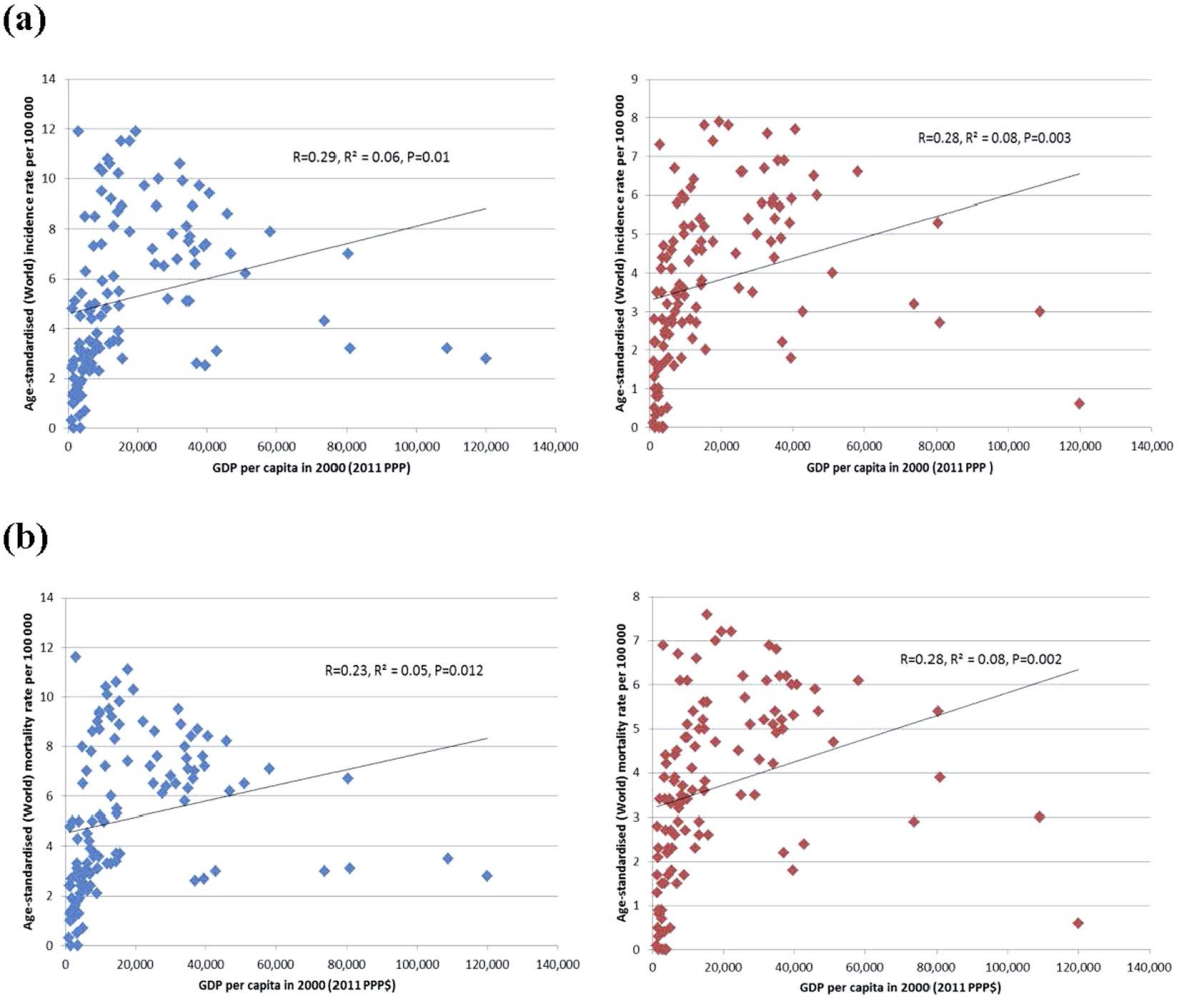
(**a**) Correlation between age-standardised mortality of pancreatic cancer in 2012 and Human Development Index (HDI) in 2000 in men (left) and women (right); (**b**). Correlation between age-standardised mortality of pancreatic cancer in 2012 and Gross Domestic Product (GDP) per capita in 2000 in men (left) and women (right).

Among all countries included in this study, the distribution of HDI levels was in a narrower range between 0.34–0.95, as compared to that of the GDP per capita that varied widely across countries. The incidence of pancreatic cancer increased with higher levels of HDI in men (r^2^ = 0.43, r = 0.66) and women (r^2^ = 0.49, r = 0.70) (Fig. 1a), and to a lesser extent this finding was observed for its correlation with GDP per capita (r^2^ = 0.08, r = 0.29 and r^2^ = 0.08, r = 0.28, all p < 0.05 for men and women, respectively) (Fig. 1b). For mortality, similar positive correlations with socioeconomic development were also observed for both HDI (r^2^ = 0.45, r = 0.67 for men and r^2^ = 0.52, r = 0.72 for women) and GDP per capita (r^2^ = 0.05, r = 0.23 for men and r^2^ = 0.08, r = 0.28, for women, all p < 0.05) (Fig. 2a and b).

### Trends in incidence and mortality from pancreatic cancer

Among men, there were a total of 10 countries with increasing incidence trends and other countries with stable incidence; whilst for women, there were 14 countries with increasing incidence. For mortality, there were 10 and 15 countries with increasing trend mortality trends in men and women, respectively. We highlighted the countries with more prominent AAPCs according to the respective continents (Supplementary Figures 1a and 1b).

#### Latin America and the Caribbean

**Figure 3:**
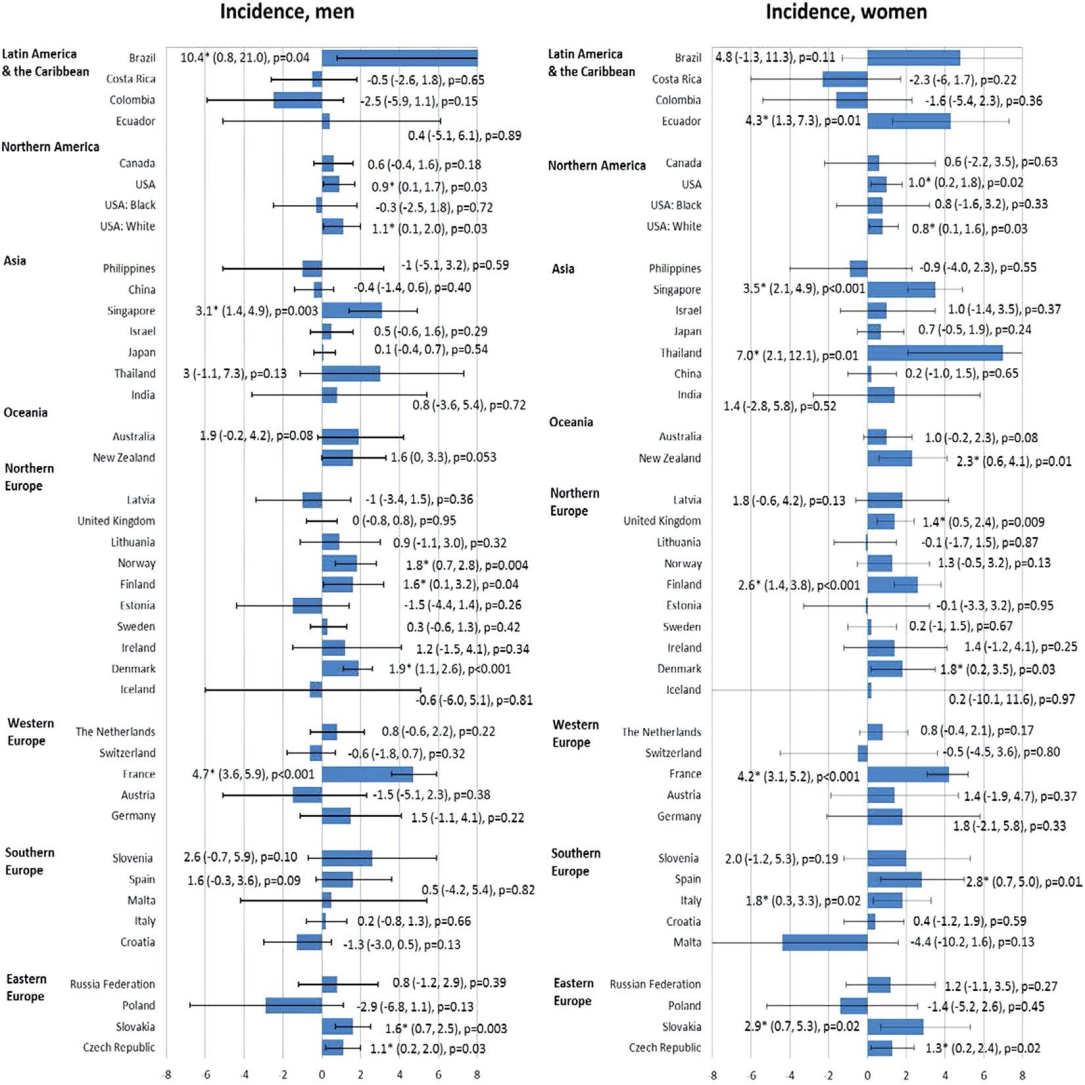
The Average Annual Percentage Change (AAPC) of pancreatic cancer incidence in men (left) and women (right) (1998–2007).

**Figure 4:**
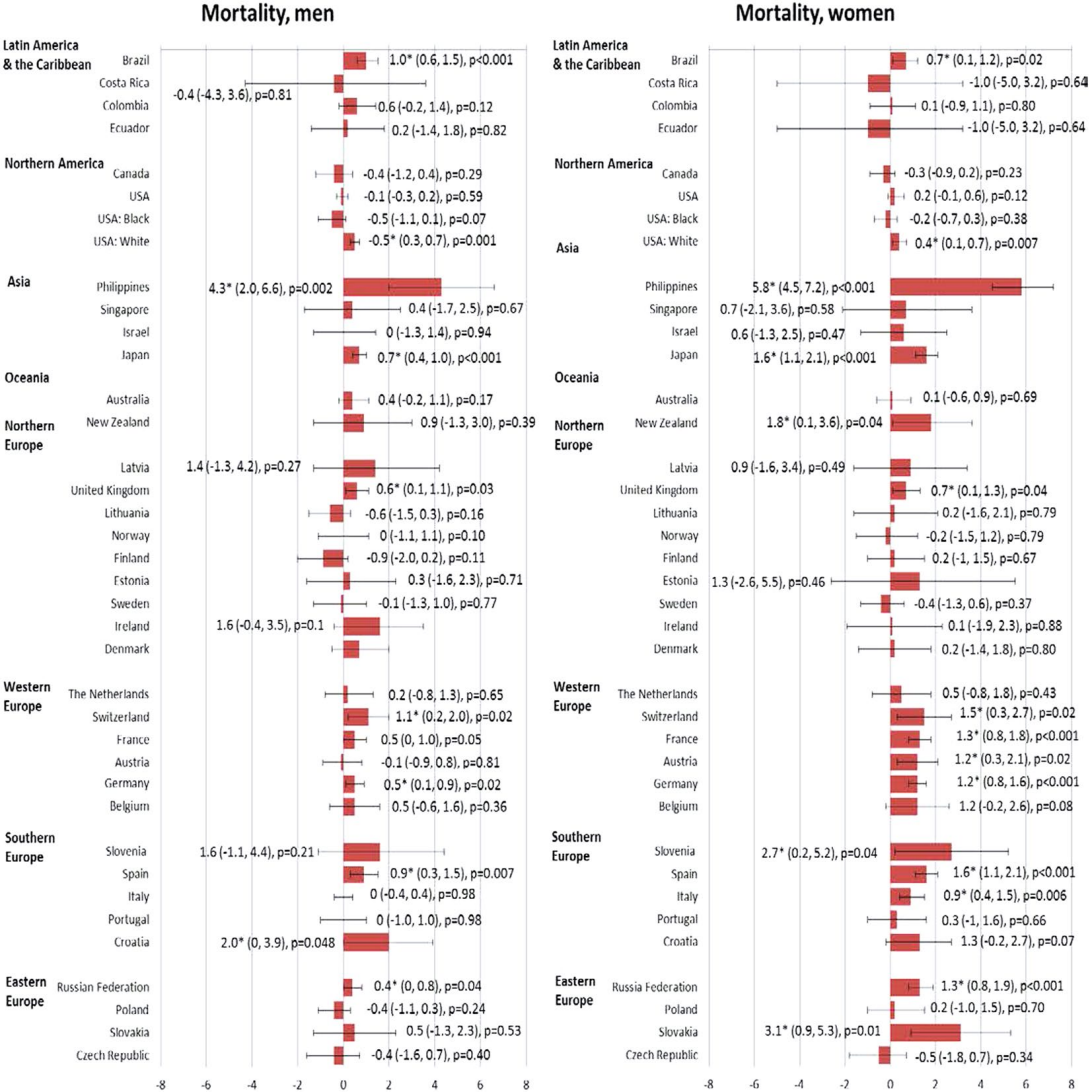
The Average Annual Percentage Change (AAPC) of pancreatic cancer mortality in men (left) and women (right) (1998–2007).

Brazil reported an increasing incidence in male (AAPC = 10.4, 95% C.I. = 0.8, 21) and increased mortality in both male (AAPC = 1.0, 95% C.I. = 0.6, 1.5) and female populations (AAPC = 0.7, 95% C.I. = 0.1, 1.2) (Figs 3 and 4). A rise in incidence was observed in Ecuador (AAPC = 4.3, 95% C.I. = 1.3, 7.3) in women.

#### Northern America

An increase in incidence was observed in male (AAPC = 0.9, 95% C.I. = 0.1, 1.7) and female (AAPC = 1.0, 95% C.I. = 0.2, 1.8) Americans. Similar trends were observed for White Americans (AAPC = 1.1, 95% C.I. = 0.1, 2.0 [men]; AAPC = 0.8, 95% C.I. = 0.1, 1.6 [women]). All other trends were stable.

#### Asia

Singapore had an increase in incidence in men (AAPC = 3.1, 95% C.I. = 1.4, 4.9) and women (AAPC = 3.5, 95% C.I. = 2.1, 4.9), whilst Thailand reported an incidence increase in female (AAPC = 7, 95% C.I. = 2.1, 12.1). The Philippines (AAPC = 4.3, 95% C.I. = 2, 6.6 [men]; AAPC = 5.8, 95% C.I. = 4.5, 7.2 [women]) and Japan (AAPC = 0.7, 95% C.I. = 0.4, 1 [men]; AAPC = 1.6, 95% C.I = 1.1, 2.1 [women]) had a significant increase in mortality rates.

#### Oceania

There was a slight increase in mortality in New Zealand among women (APCC = 1.8, 95% C.I. = 0.1, 3.6). All other incidence and mortality trends were stable.

#### Northern Europe

Norway had an increase in incidence among men (AAPC = 1.8, 95% C.I. = 0.7, 2.8), whereas the United Kingdom (AAPC = 1.4, 95% C.I. = 0.5, 2.4) reported an increase in incidence among women. Finland and Denmark reported an increase in incidence among men and women. For mortality, only the United Kingdom showed an increase in men (AAPC = 0.6, 95% C.I. = 0.1, 1.1) and women (AAPC = 0.7, 95% C.I. = 0.1, 1.3). All other incidence and mortality trends were stable.

#### Western Europe

France showed an increase in incidence among men (AAPC = 4.7, 95% C.I. = 3.6, 5.9) and women (AAPC = 4.2, 95% C.I. = 3.1, 5.2). Switzerland reported an increase in mortality in men (AAPC = 1.1, 95% C.I. = 0.2, 2). Switzerland (AAPC = 1.5, 95% C.I. = 0.3, 2.7), France (AAPC = 1.3, 95% C.I. = 0.8, 1.8), Austria (AAPC = 1.2, 95% C.I. = 0.3, 2.1) and Germany (AAPC = 1.2, 95% C.I. = 0.8, 1.6) showed increasing trends in mortality among women.

#### Southern Europe

Spain (AAPC = 2.8, 9.5% C.I. = 0.7, 5) and Italy (AAPC = 1.8, 95% C.I. = 0.3, 3.3) had increases in incidence among women. Croatia (AAPC = 2.0, 95% C.I. = 0, 3.9) and Spain (AAPC = 0.9, 95% C.I. = 0.3, 1.5) showed an increase in mortality in men, whilst Slovenia (AAPC = 2.7, 95% C.I. = 0.2, 5.2), Spain (AAPC = 1.6, 95% C.I. = 1.1, 2.1) and Italy (AAPC = 0.9, 95% C.I. = 0.4, 1.5) showed a rise in mortality among women.

#### Eastern Europe

An increasing incidence trend was observed for Slovakia (AAPC = 1.6, 95% C.I. = 0.7, 2.5 [men]; AAPC = 2.9, 95% C.I. = 0.7, 5.3 [women]) and Czech Republic (AAPC = 1.1, 95% C.I. = 0.2, 2 [men]; AAPC = 1.3, 95% C.I. = 0.2, 2.4). The Russian Federation had increases in mortality trends in male (AAPC = 0.4, 95% C.I. = 0, 0.8) and female (AAPC = 1.3, 95% C.I. = 0.8, 1.9) population. Slovakia showed a prominent rise in mortality among women (AAPC = 3.1, 95% C.I. = 0.9, 5.3).

Taking into account these temporal trends, the most remarkable observation included the increase in incidence in Thailand and France in both men and women; Brazil in men and Ecuador in women. There were drastic rises in mortality rates in the Philippines and Croatia in men, and very substantial increase in mortality in the Philippines, Slovenia and Slovakia in women.

## Discussion

This study presented a comprehensive epidemiological analysis of the global profiles of pancreatic cancer incidence and mortality based on high quality data. Evaluating and analyzing the patterns and temporal trends of this cancer could identify high-risk populations, delineate the extent of preventive strategies implemented, and eventually provide further insights into disease etiology. As of 2012, North America, various parts of Europe and Australia/New Zealand suffered from the highest incidence and mortality in both genders. South-Central Asia, Middle Africa, Eastern Africa and Western Africa reported the lowest incidence and mortality in both men and women. The highest incidence to mortality ratio was found in Australia/New Zealand in both genders. It was found that countries with higher levels of HDI and GDP per capita reported higher incidence and mortality rates of pancreatic cancer. The coefficients of determination (R^2^) of HDI and GPD per capita for the incidence and mortality were high. Most countries included in the analysis presented insignificant changes in incidence and mortality trends, with relatively wide 95% confidence intervals. There were only four and five countries, respectively, that showed the presence of joinpoints for incidence and mortality trends in the statistical analysis. No variation in the rate of change was observed in most countries.

The results from this study in general corroborated the findings of previous observations^4, 5, 35^, where the incidence were higher in antipodal countries than those located on or close to the equator. As highlighted by Maisonneuve and Lowenfels^4^, the risk of pancreatic cancer could be higher in countries exposed to lower levels of solar radiation – which were related to sunlight and ultraviolet radiation^36^. Another explanation for the higher incidence of pancreatic cancer in Western countries could be due to the ageing population and recent changes in lifestyle factors, which were the strongest risk factor for pancreatic cancer^6–12, 37^. These risk factors could also account for the higher incidence and mortality of pancreatic cancer in countries with higher socioeconomic development, since they are in general more prevalent in more developed regions. Yet these remain speculative as the findings of this study did not provide any data to support these possible explanations. It should also be acknowledged that there exists a possible difference between grouping antipodal countries vs. those close to the equator and developed vs. less developed countries. In addition, the higher rates observed in men could be due to their higher smoking rates^18^, and the similar magnitude of the age-standardized rates between incidence and mortality could be attributed to the relatively poor survival rates of pancreatic cancer upon diagnostic confirmation.

It is notable from this study that none of the countries examined showed a declining incidence or mortality trend of pancreatic cancer. This observation highlighted that this cancer is still an important global health issue. Future mechanistic studies are needed to establish the association between socioeconomic development and pancreatic cancer incidence. It should also be noted that one cannot easily speculate the reasons for the increasing incidence and mortality rates in those countries found to have such trends. These may need further epidemiological analysis to address the current knowledge gaps in its potential etiology, including the substantial racial differences in its frequency, the linkage with blood groups, the possible association between pancreatic cancer and oncogenic viruses, as well as the validation of many putative gene polymorphisms^18^.

This study presented and analyzed the most up-to-date epidemiological data on pancreatic cancer using data of high validity, completeness and comparability. We also adopted figures on national mortality that fulfilled criteria attaining at least WHO-defined medium levels of coverage and completeness. The IARCs estimation methods have been further refined in more recent years to take into account the increasing availability and quality of the source data^38^. These epidemiological data could be linked to the future prospects of cancer surveillance for policy-makers and health practitioners. Nevertheless, some limitations should be addressed. Firstly, failure or under-reporting of cancer diagnosis could lead to bias in cancer registration especially in relatively less-developed nations. Figures in regional cancer registries could be underestimated owing to limited local facilities. On the contrary, in countries where estimates were based on a single cancer registry in more urbanized, resource privileged areas, the presented figures could be an overestimation if the countries consist of extensive rural populations. In addition, only one-third and one-fifth of the world’s countries, respectively, reported incidence and mortality data of high quality. For instance, the death certificates cover only 30% of deaths in the world population, and this proportion could be unequally distributed. While in some industrialized countries almost all death statistics are based on death certificates, the proportion is very low in developing nations where a large proportion of death cause is based on verbal autopsy or indirect estimation methods. As a result, the incidence and mortality data are constrained with respect to geographical coverage, in particular the resource-deprived countries. One should also interpret the findings with caution, as attribution bias might exist, particularly in countries with relatively deprived infrastructure on disease reporting. Finally, this descriptive analysis did not take into account the epidemiology of risk factors of pancreatic cancer when comparisons were made among countries, and the inherent limitations of ecological studies should be noted^39^.

In summary, the incidence and mortality rates of pancreatic cancer increased in many countries. With population growth, clinicians and policy-makers might expect a further substantial rise in its global health burden – particularly countries with socioeconomic development. Hence, more healthcare resources are needed to cope with the treatment and follow-up consultations of patients diagnosed with the cancer, in particular for the more resource-deprived countries and regions with fewer preventive strategies such as smoking cessation and obesity-reduction programmes. Future studies should explore the underlying reasons for these epidemiological trends, which could offer further insights into the specific etiological factors of pancreatic cancer.

## Electronic supplementary material

Supplementary Figures
