# Supplementary material for: Global temporal patterns of pancreatic cancer and association with socioeconomic development
Source: Sci Rep. 2017 Jun 9;7:3165. doi: 10.1038/s41598-017-02997-2 (PMC5466634; doi:10.1038/s41598-017-02997-2)

Global patterns and temporal trend of pancreatic cancer: a joinpoint regression analysis

Martin CS Wong MD, MPH[1, 2], *, Johnny JY Jiang MD, PhD[3], Miaoyin Liang MPH[1], Yuan Fang PhD[1], Ming Sze Yeung BSc (Hons)[1], Joseph JY Sung MD, PhD[2, 4, 5]

1. School of Public Health and Primary Care, Faculty of Medicine, Chinese University of Hong Kong. Prince of Wales Hospital, Shatin, New Territories, Hong Kong

2. Institute of Digestive Disease, Faculty of Medicine, Chinese University of Hong Kong

3. Chinese Academy of Medical Sciences and Peking Union Medical College, China

4. Department of Medicine and Therapeutics, Faculty of Medicine, Chinese University of Hong Kong

5. State Key Laboratory of Digestive Disease, Faculty of Medicine, Chinese University of Hong Kong

Keywords: pancreatic cancer; global trend; incidence; mortality; socioeconomic development

Correspondence:

Martin C.S. Wong BMedSc (Hons), MSc (Hons), MBChB, MD, MPH, MBA, FRSPH, FRSP,

FRIPH, FRACGP, FHKCFP, DCH, FHKAM, FHKAN

Professor of Family Medicine and Primary Healthcare, JC School of Public Health and Primary Care, Faculty of Medicine, The Chinese University of Hong Kong

Consultant (Honorary), Department of Family Medicine, Hospital Authority

Tel: (852) 2252 8782 **Fax**: (852) 2606 3500; **Email**: wong_martin@cuhk.edu.hk

Address: 4/F, School of Public Health, Prince of Wales Hospital, Shatin, N.T., Hong Kong

Supplementary Figure 1a Findings from the joinpoint regression analysis of the global incidence rates of pancreatic cancer (Left: Male, Right: Female)

1) Latin America and the Caribbean

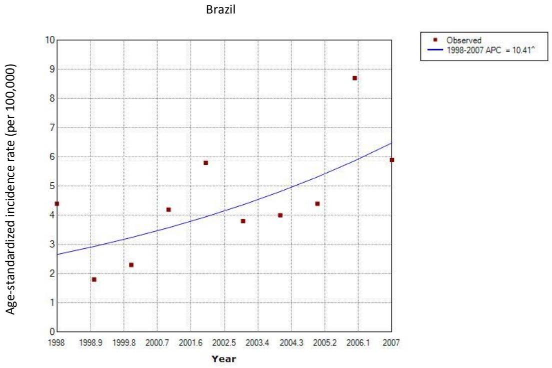

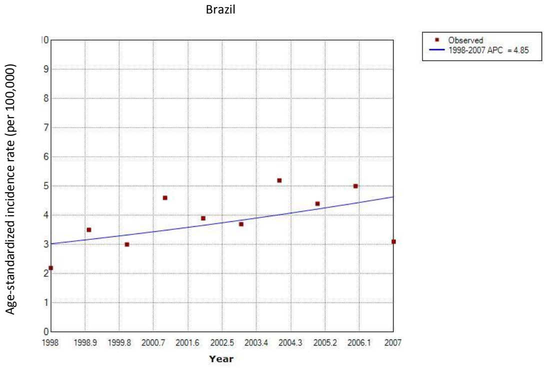

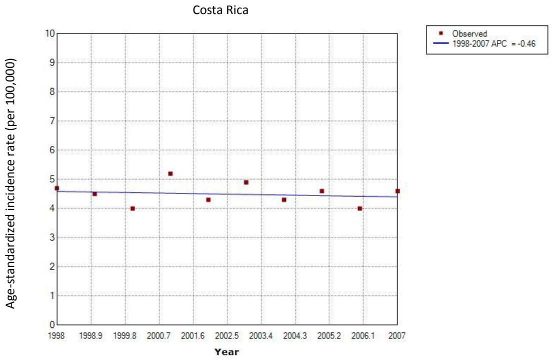

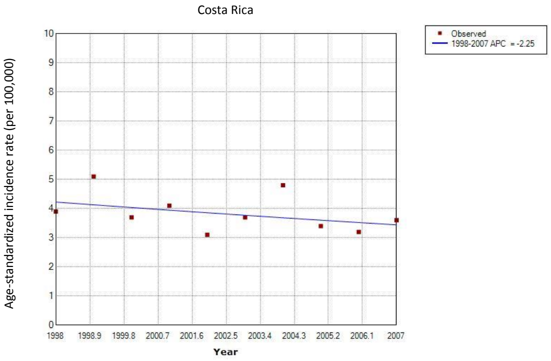

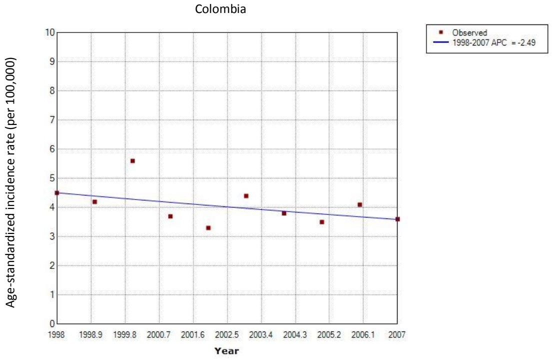

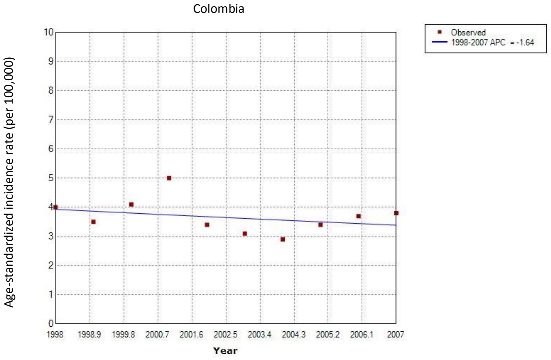

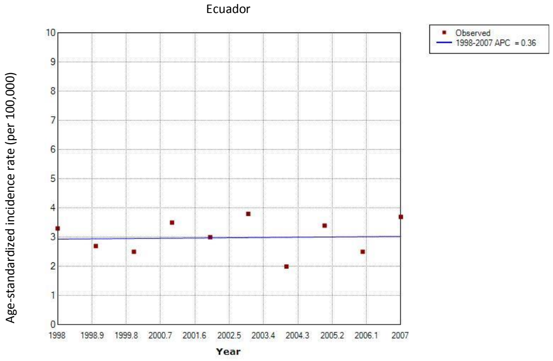

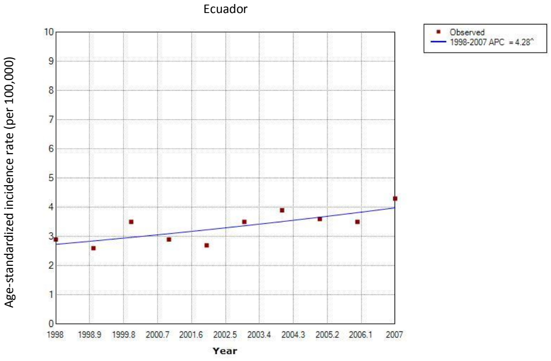

2) Northern America

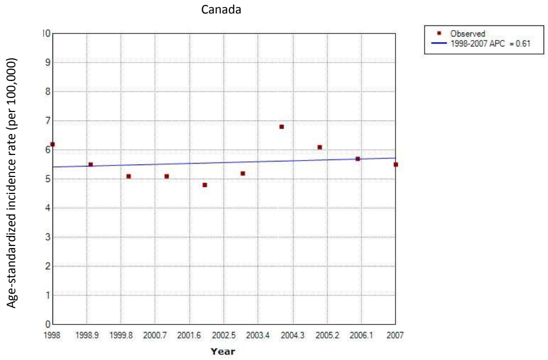

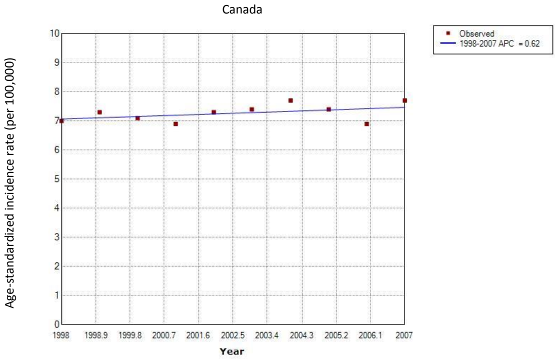

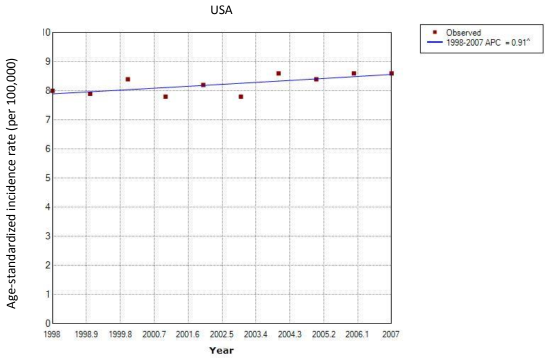

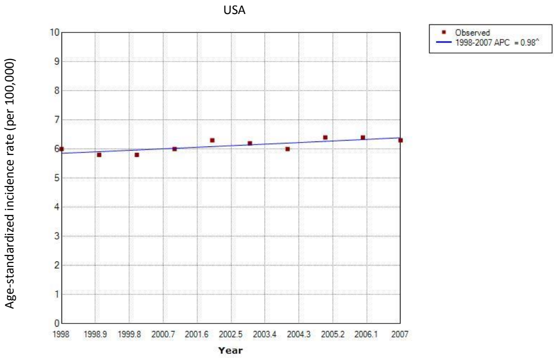

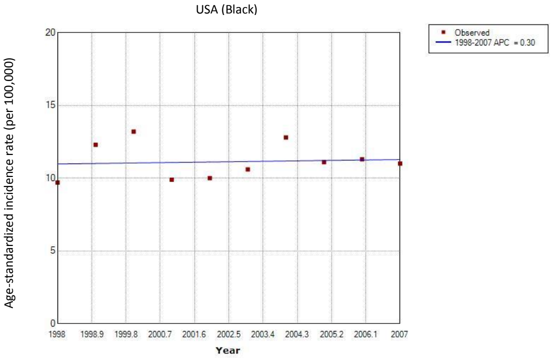

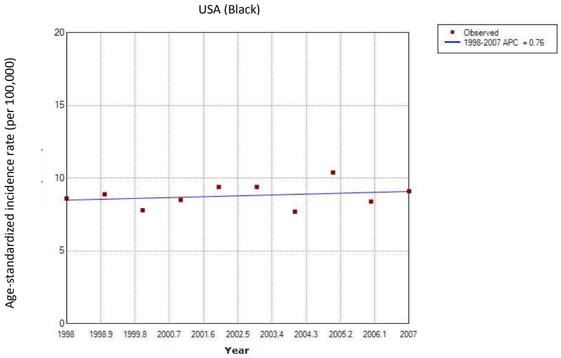

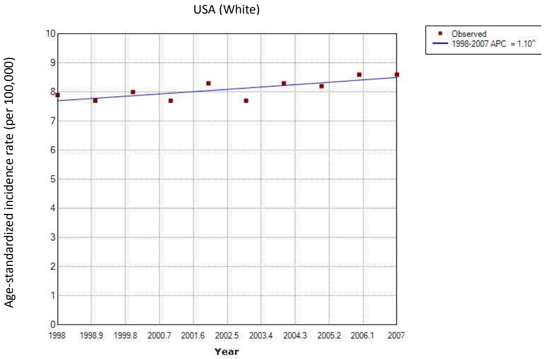

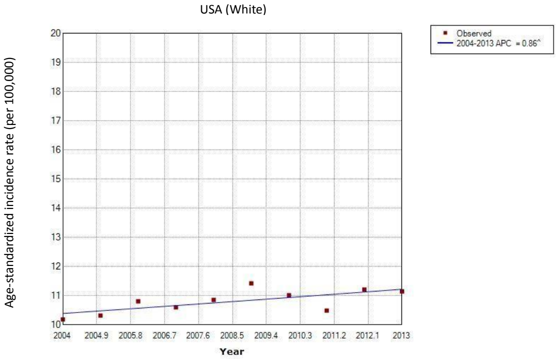

3) Asia

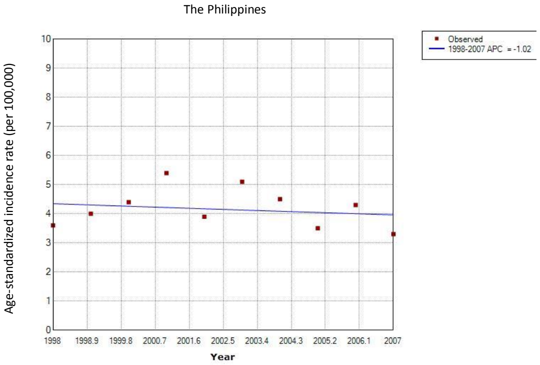
The Philippines
Observed
1998-2007 APC = -1.02
Age-standardized incidence rate (per 100,000)
Year

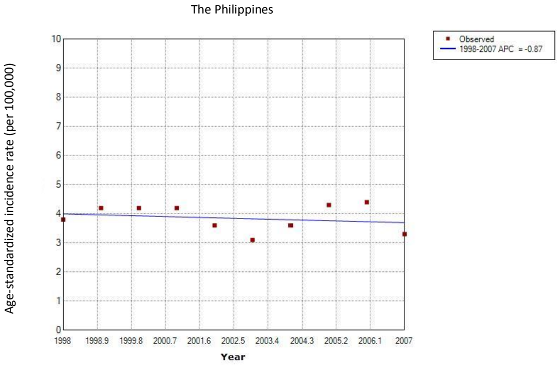
The Philippines
Observed
1998-2007 APC = -0.87
Age-standardized incidence rate (per 100,000)
Year

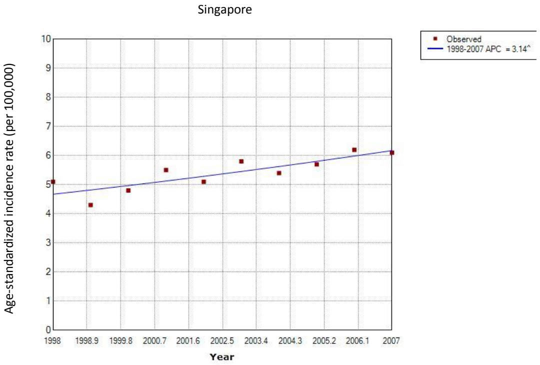
Singapore
Observed
1998-2007 APC = 3.14^
Age-standardized incidence rate (per 100,000)
Year

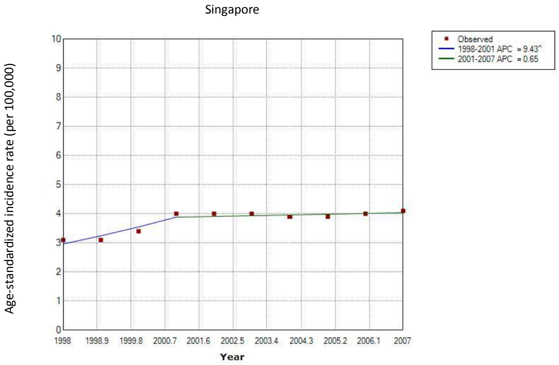
Singapore
Observed
1998-2001 APC = 9.43^
2001-2007 APC = 0.65
Age-standardized incidence rate (per 100,000)
Year

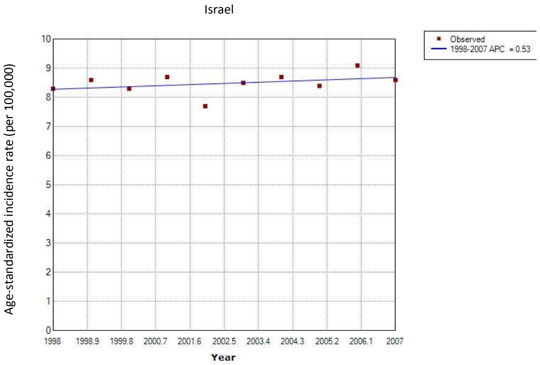
Israel
Observed
1998-2007 APC = 0.53
Age-standardized incidence rate (per 100,000)
Year

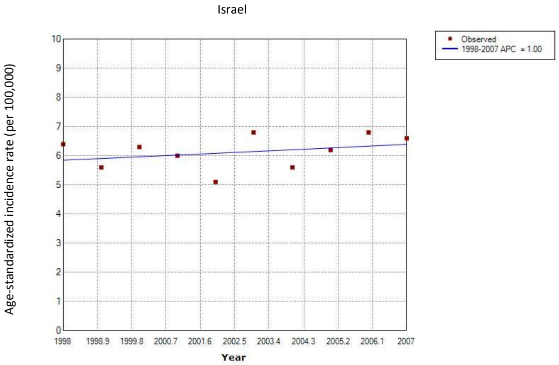
Israel
Observed
1998-2007 APC = 1.00
Age-standardized incidence rate (per 100,000)
Year

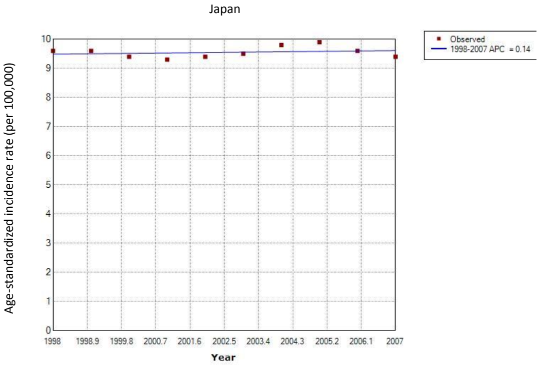
Japan
Observed
1998-2007 APC = 0.14
Age-standardized incidence rate (per 100,000)
Year

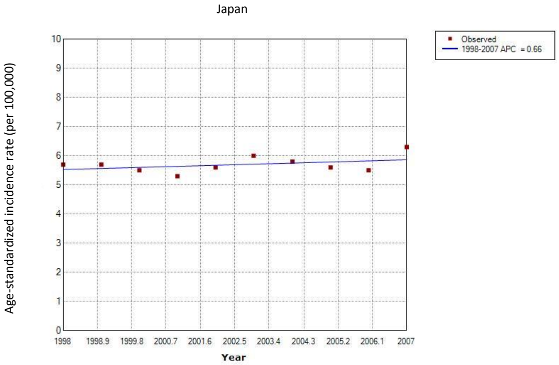
Japan
Observed
1998-2007 APC = 0.66
Age-standardized incidence rate (per 100,000)
Year

Thailand

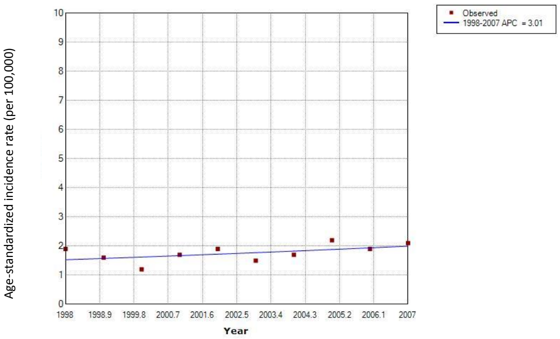

Thailand

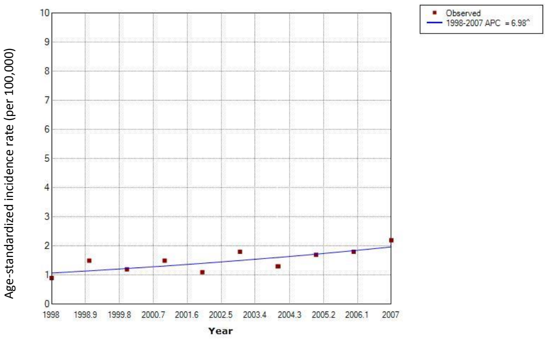

China

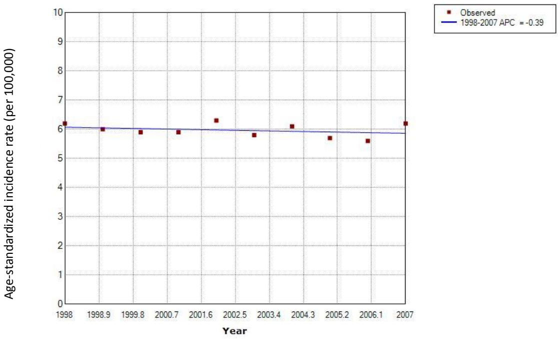

China

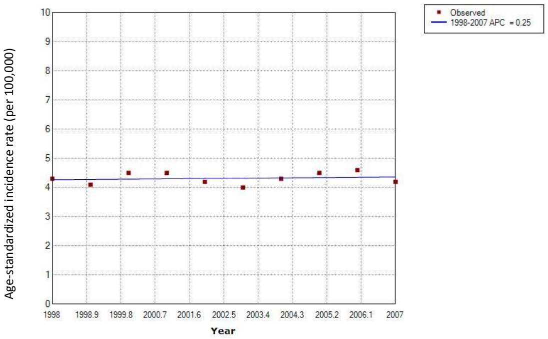

India

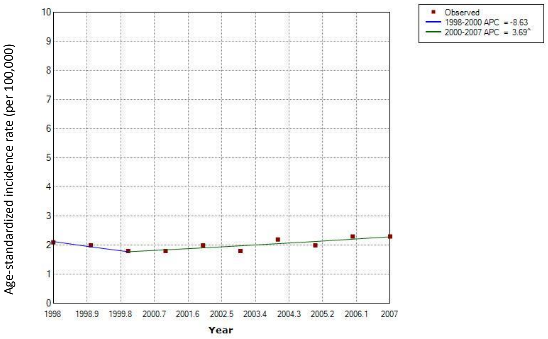

India

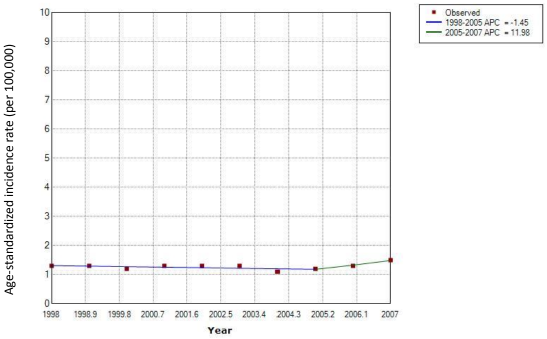

4) Oceania

Australia

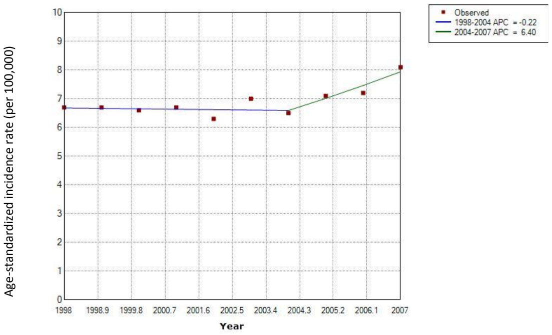

Australia

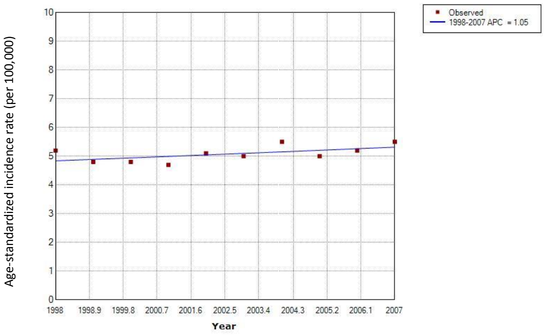

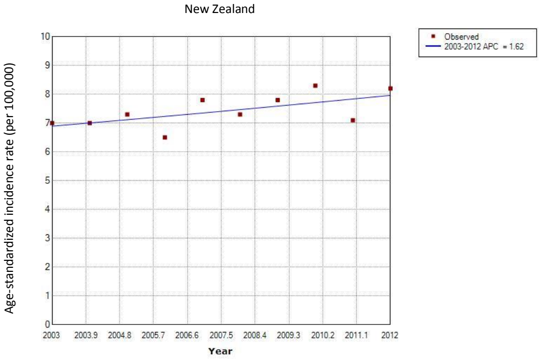

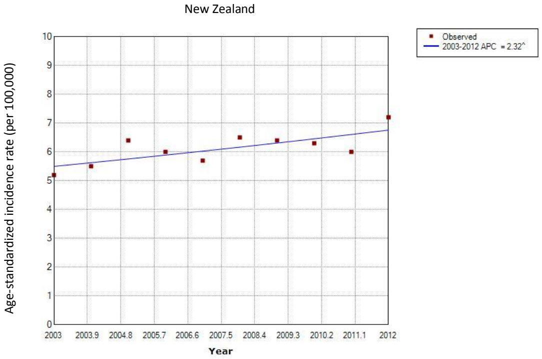

5) Northern Europe

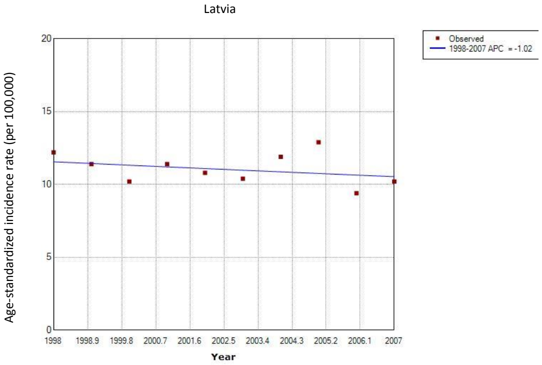

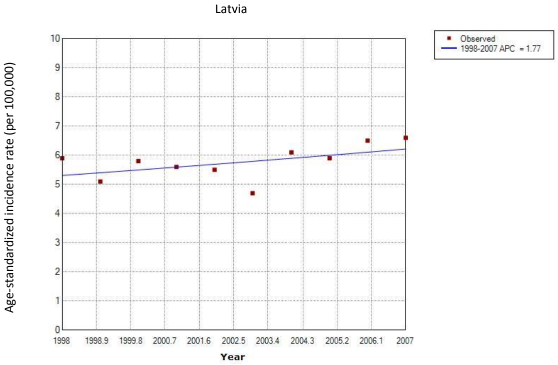

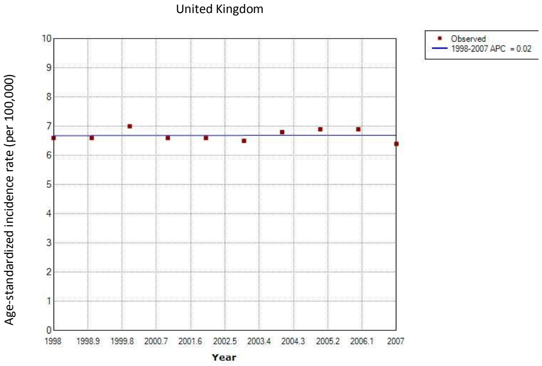

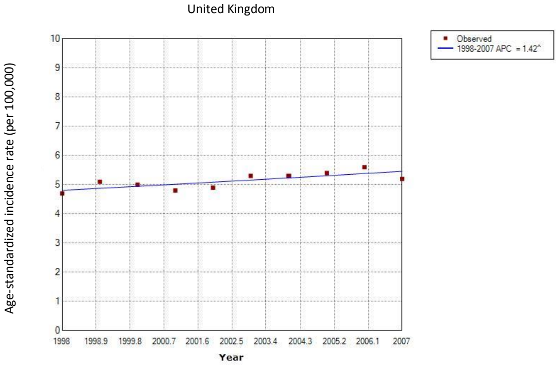

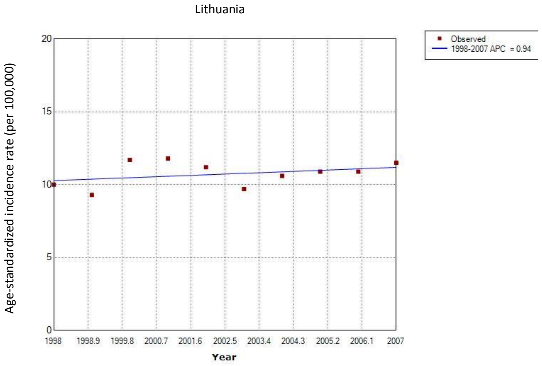

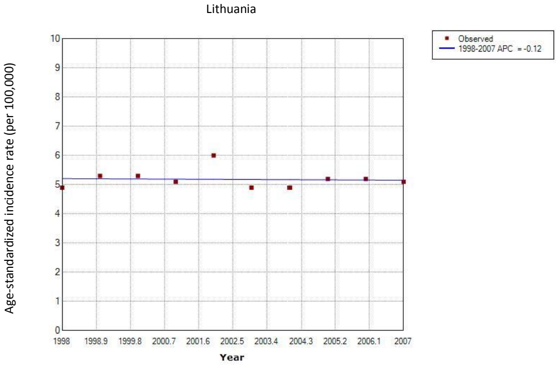

Norway

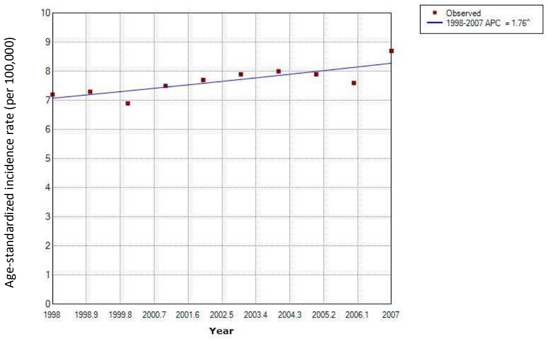

Norway

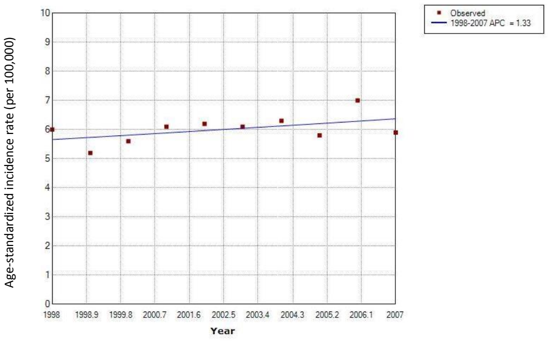

Finland

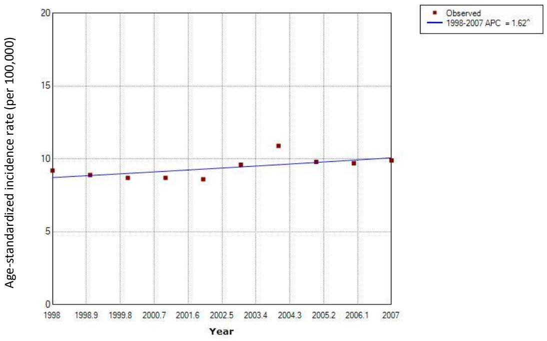

Finland

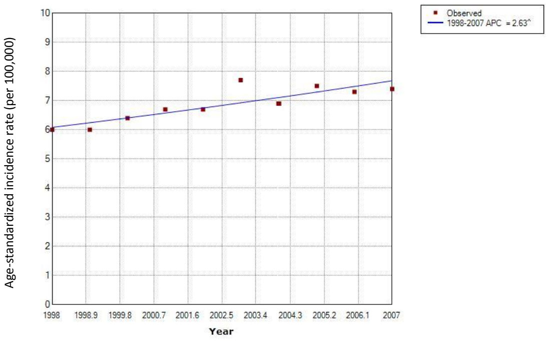

Estonia

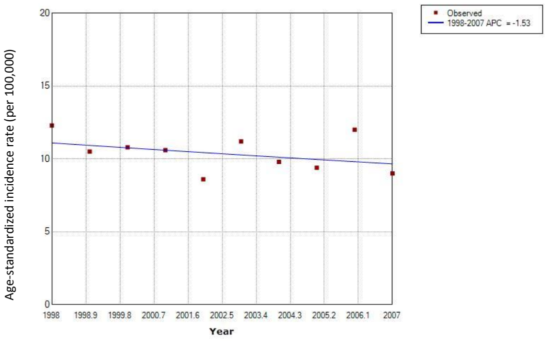

Estonia

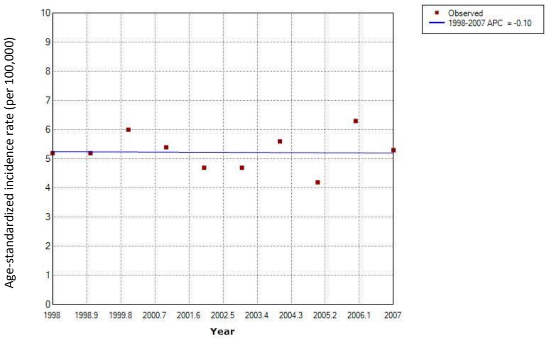

Sweden

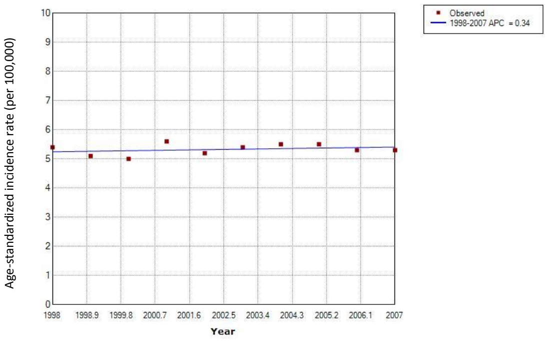

Sweden

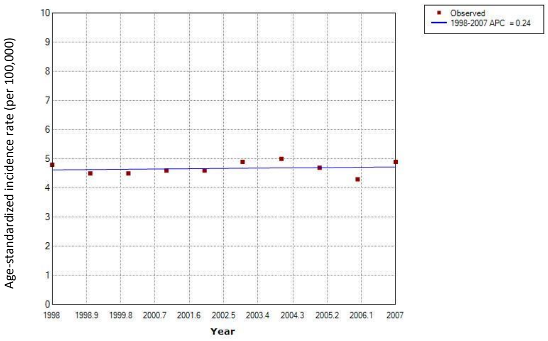

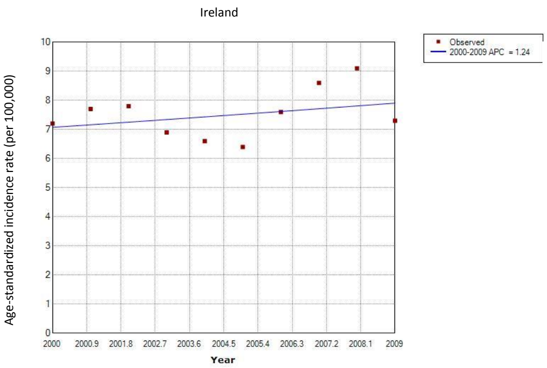

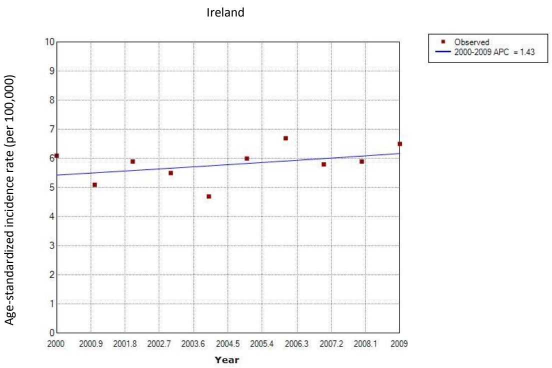

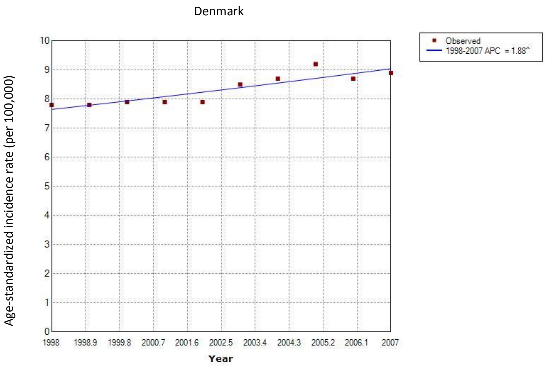

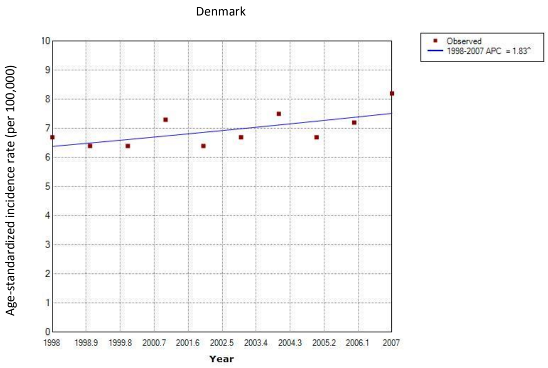

1) Western Europe

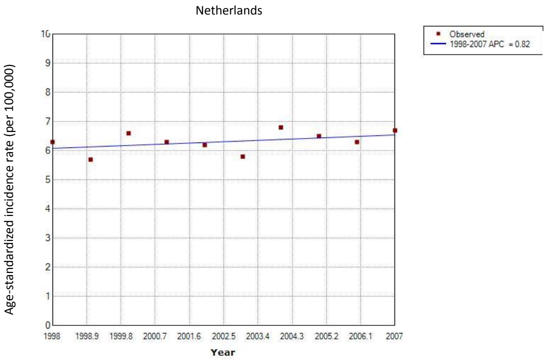

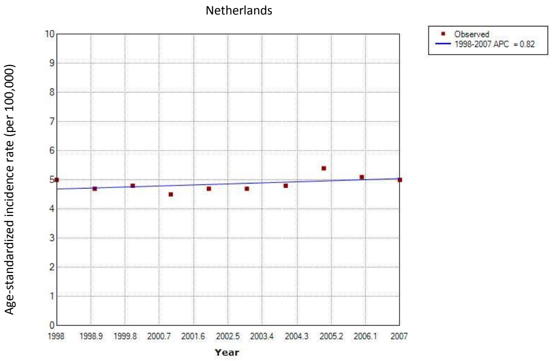

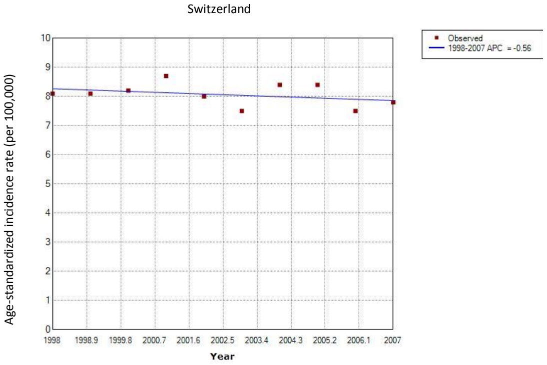

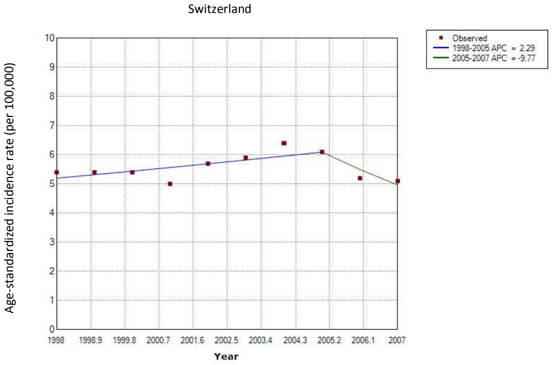

France

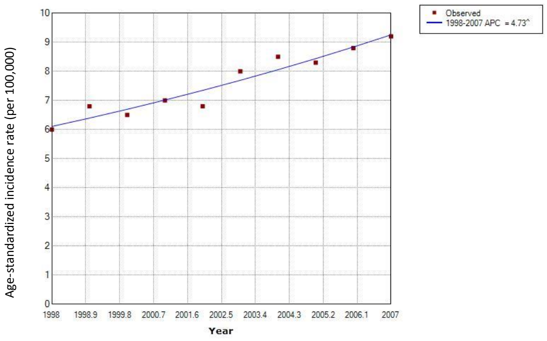

France

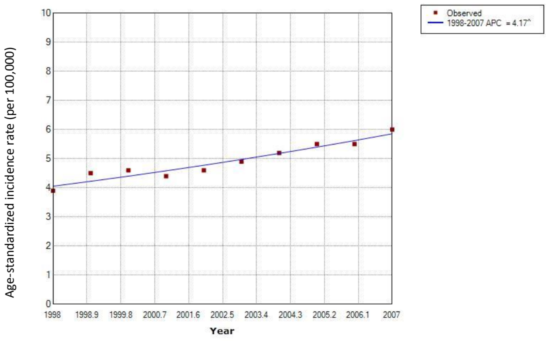

Austria

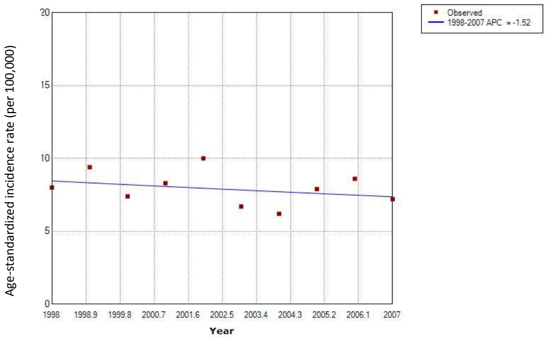

Austria

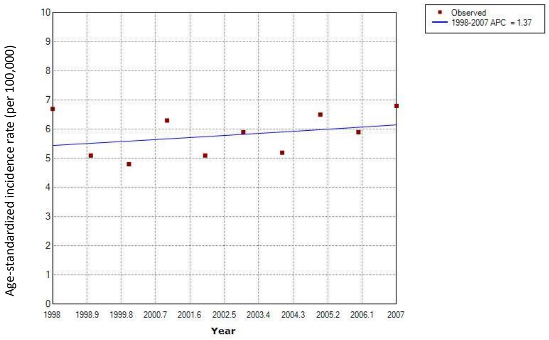

Germany

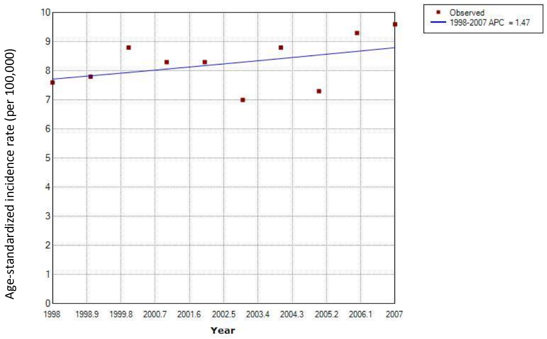

Germany

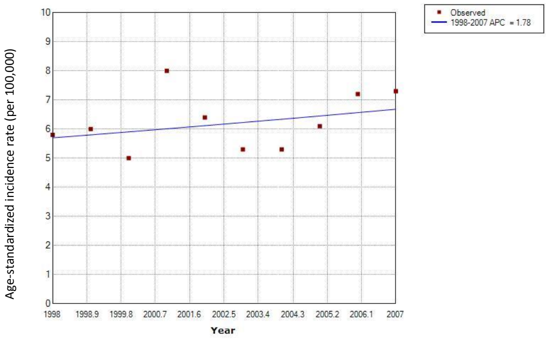

7) Southern Europe

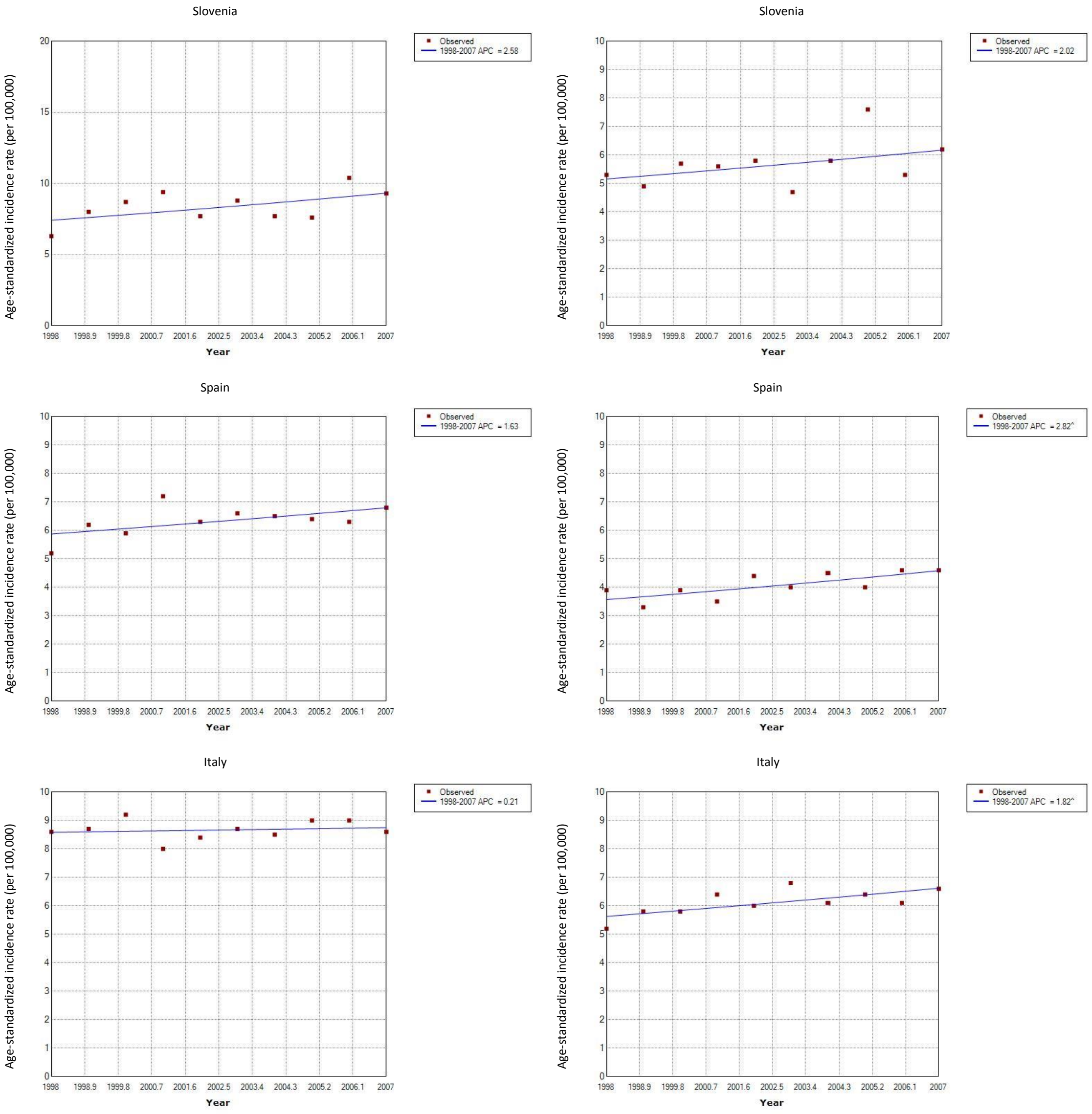

Slovenia
Observed
1998-2007 APC = 2.58
Slovenia
Observed
1998-2007 APC = 2.02
Spain
Observed
1998-2007 APC = 1.63
Spain
Observed
1998-2007 APC = 2.82^
Italy
Observed
1998-2007 APC = 0.21
Italy
Observed
1998-2007 APC = 1.82^
Age-standardized incidence rate (per 100,000)
Year
1998
1998.9
1999.8
2000.7
2001.6
2002.5
2003.4
2004.3
2005.2
2006.1
2007

Croatia

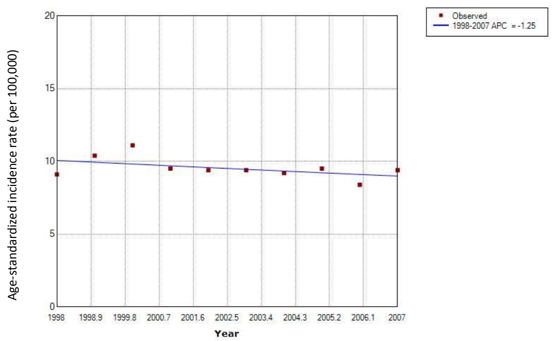

Croatia

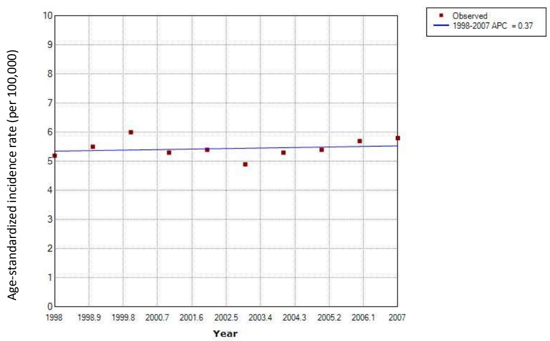

Malta

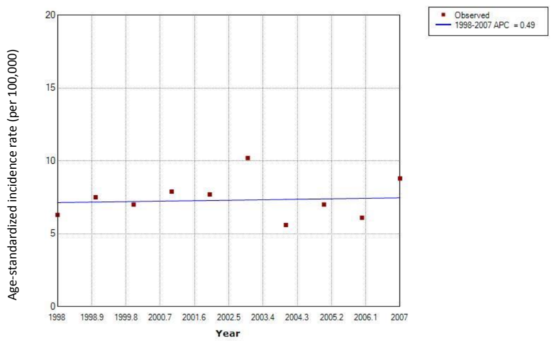

Malta

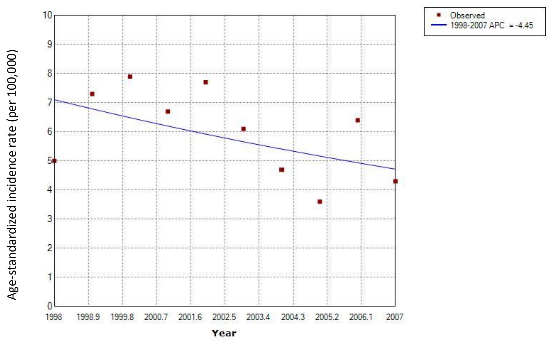

8) Eastern Europe

Russia

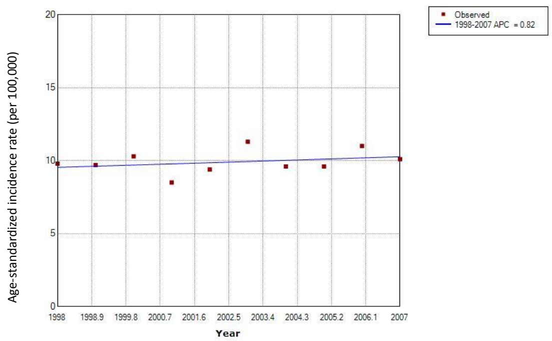

Russia

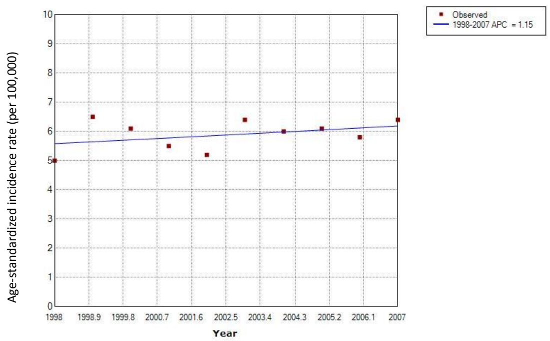

Poland

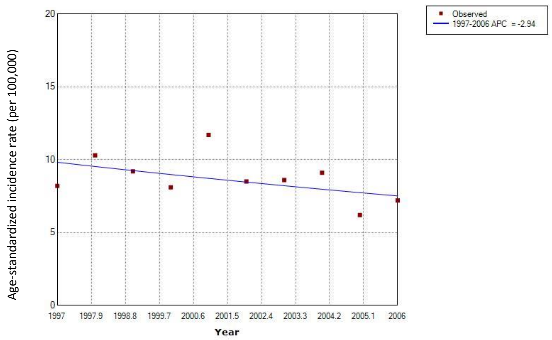

Poland

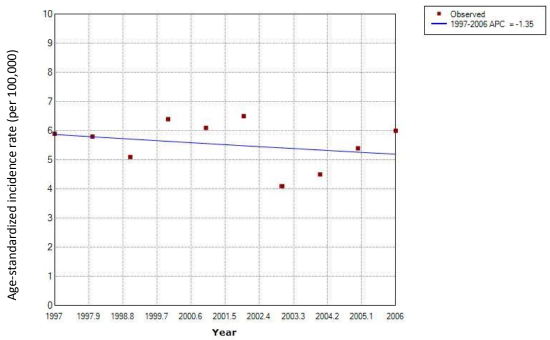

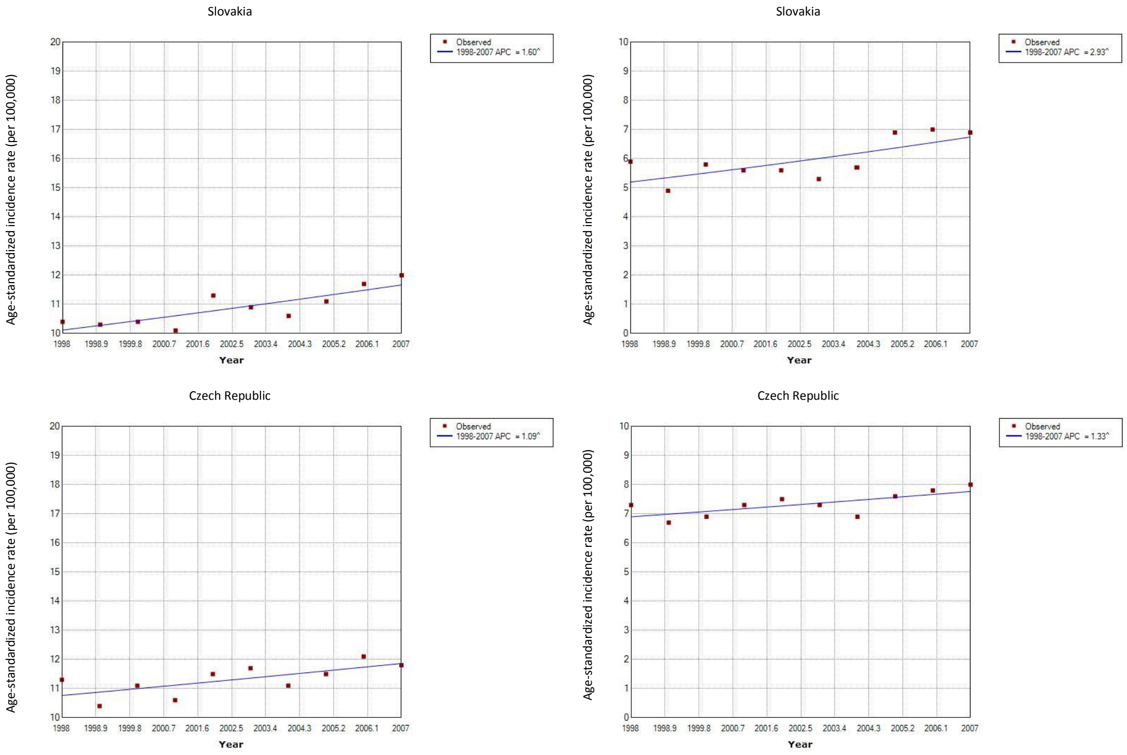

Slovakia
Observed
1998-2007 APC = 1.60^
Age-standardized incidence rate (per 100,000)
Year
Slovakia
Observed
1998-2007 APC = 2.93^
Age-standardized incidence rate (per 100,000)
Year
Czech Republic
Observed
1998-2007 APC = 1.09^
Age-standardized incidence rate (per 100,000)
Year
Czech Republic
Observed
1998-2007 APC = 1.33^
Age-standardized incidence rate (per 100,000)
Year

Supplementary Figure 1b Findings from the joinpoint regression analysis of the global mortality rates of pancreatic cancer (Left: Male, Right: Female)

1) Latin America and the Caribbean

Brazil

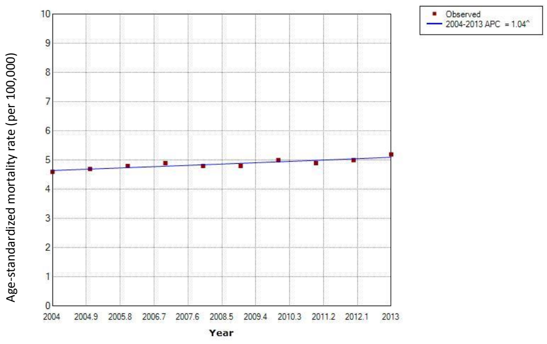

Brazil

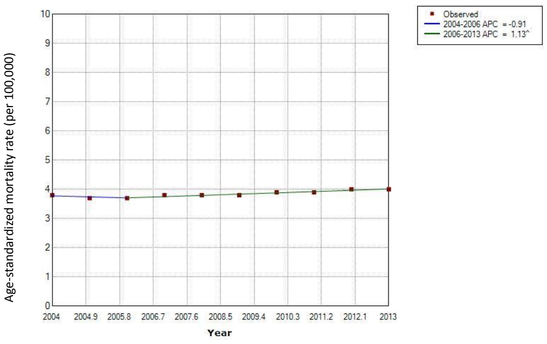

Costa Rica

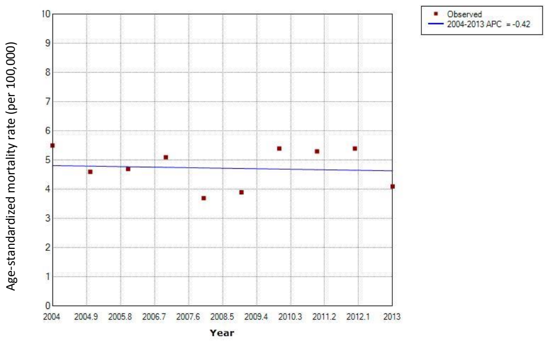

Costa Rica

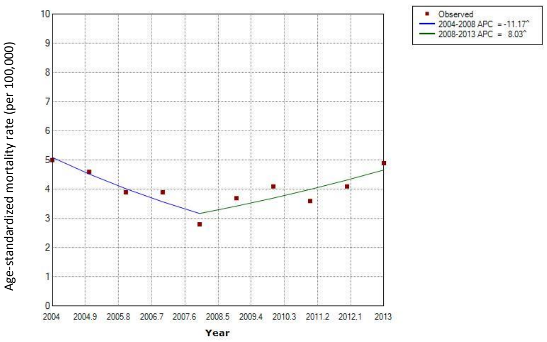

Colombia

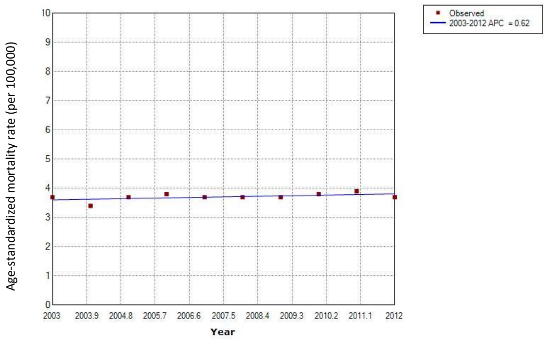

Colombia

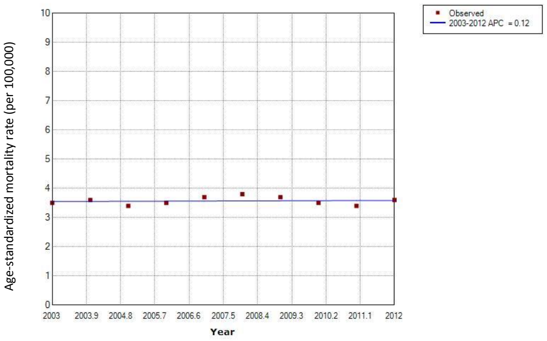

Northern America

Canada

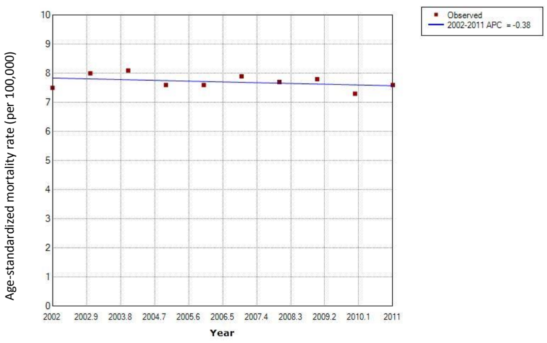

Canada

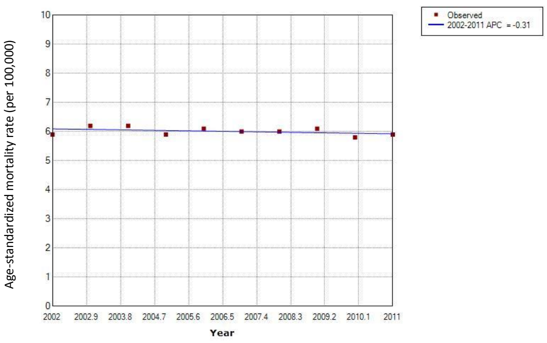

USA

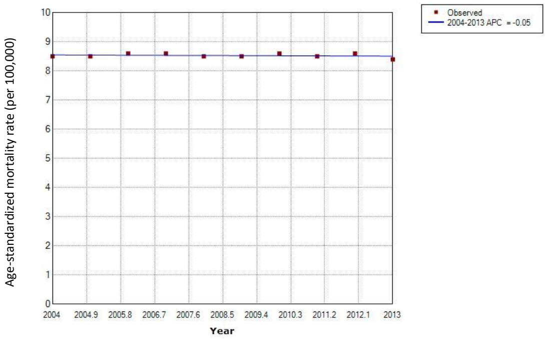

USA

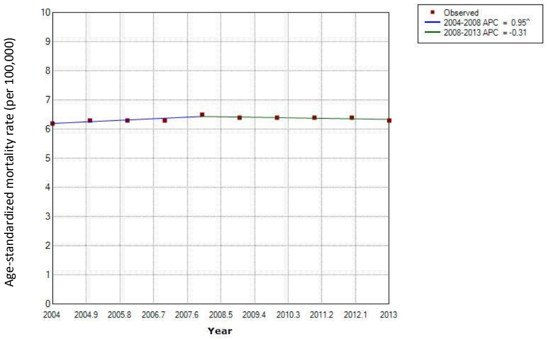

USA (Black)

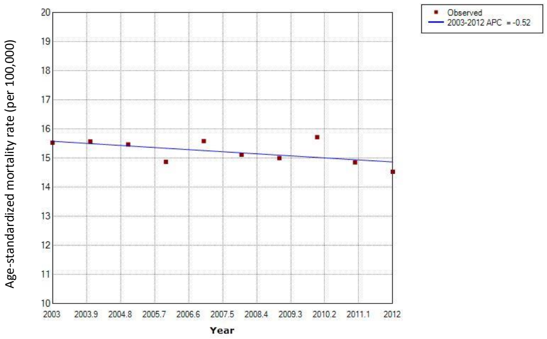

USA (Black)

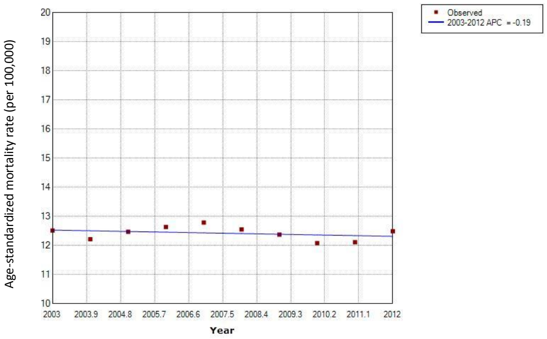

USA (White)

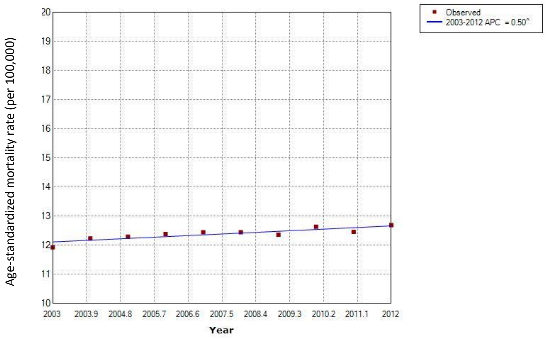

USA (White)

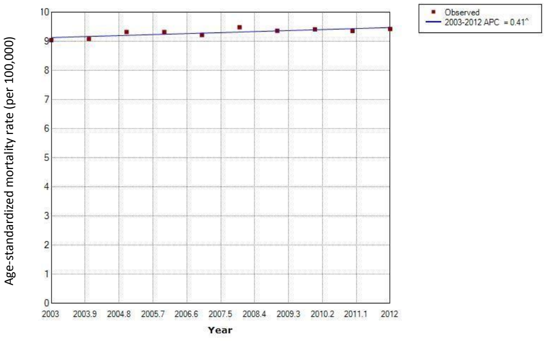

2) Asia

The Philippines

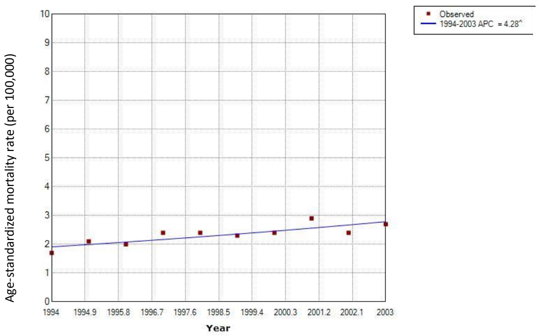

The Philippines

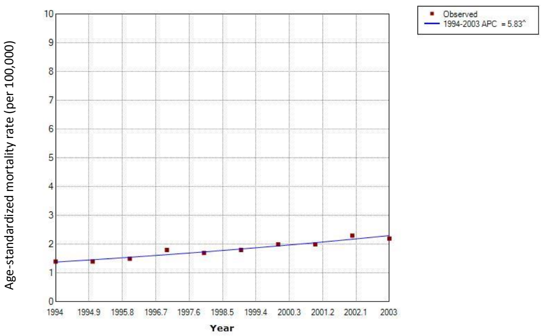

Singapore

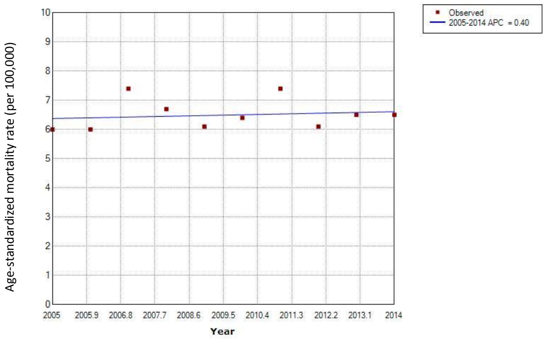

Singapore

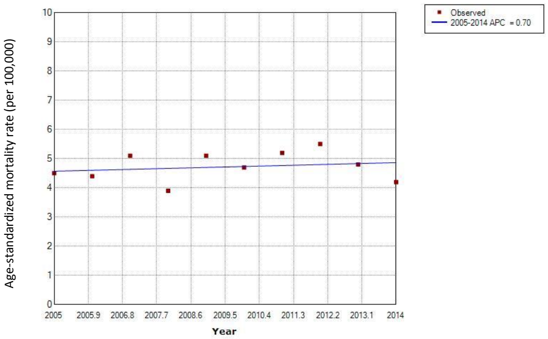

Israel

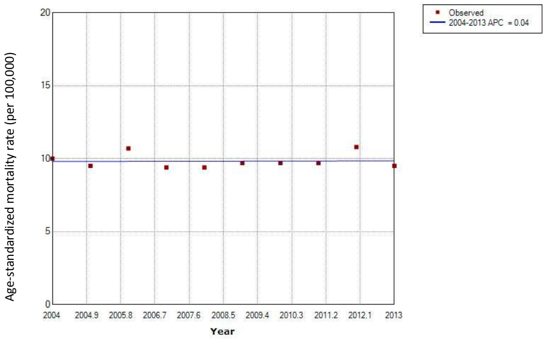

Israel

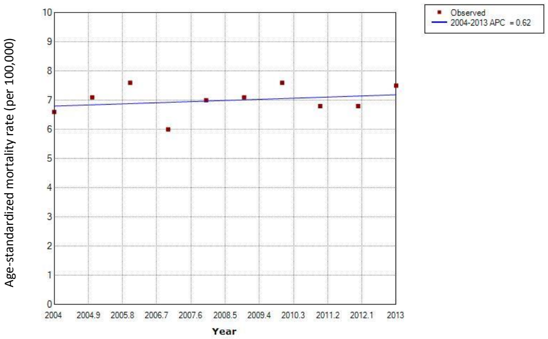

Japan

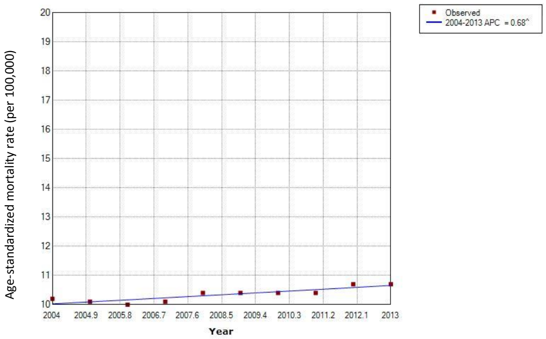

Japan

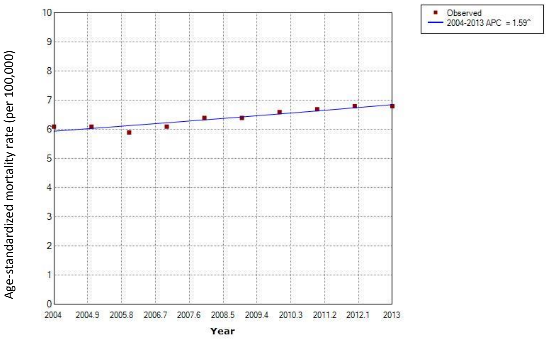

3) Oceania

Australia

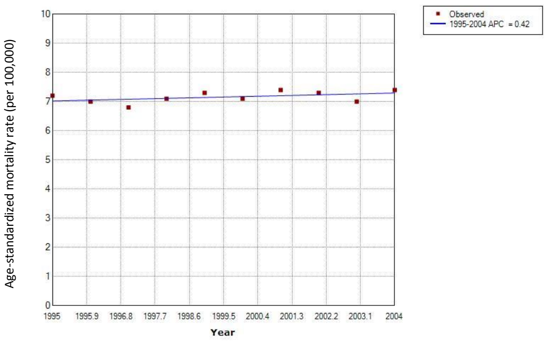

Australia

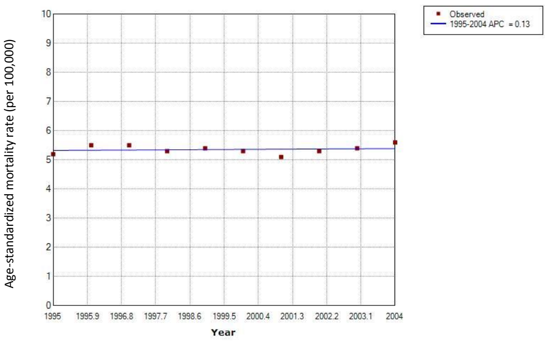

New Zealand

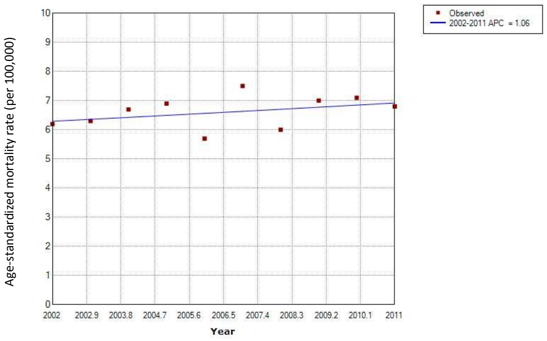

New Zealand

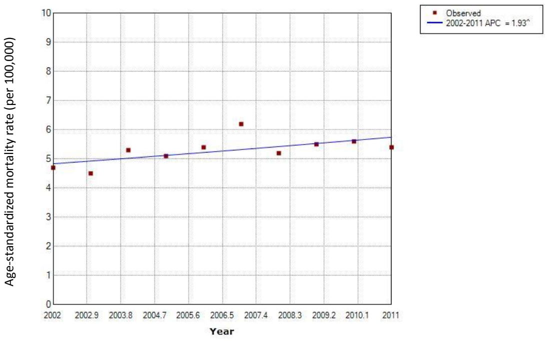

4) Northern Europe

Latvia

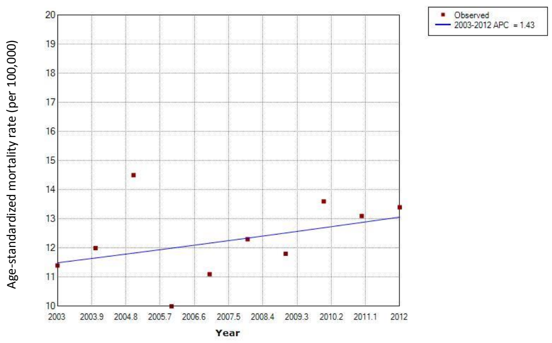

Latvia

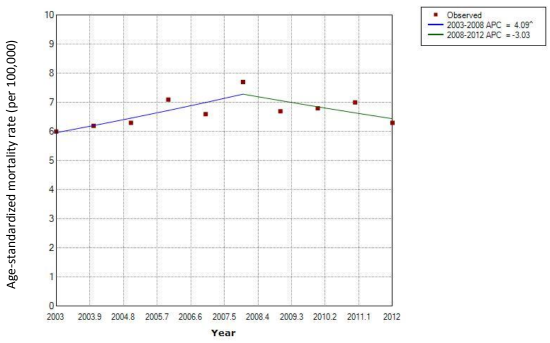

United Kingdom

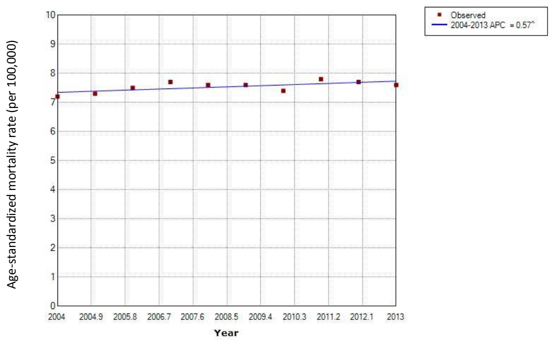

United Kingdom

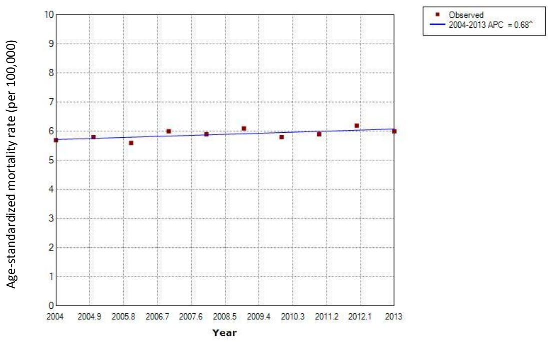

Lithuania

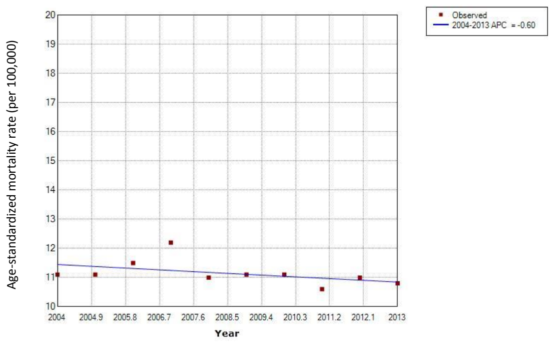

Lithuania

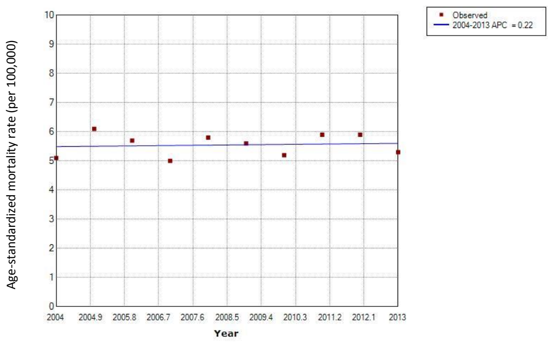

Norway

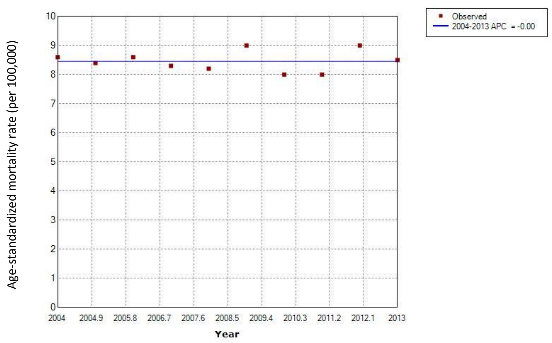

Norway

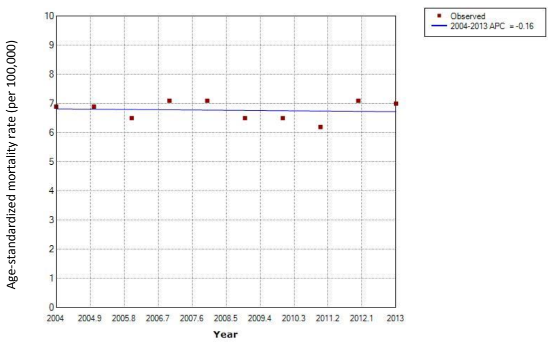

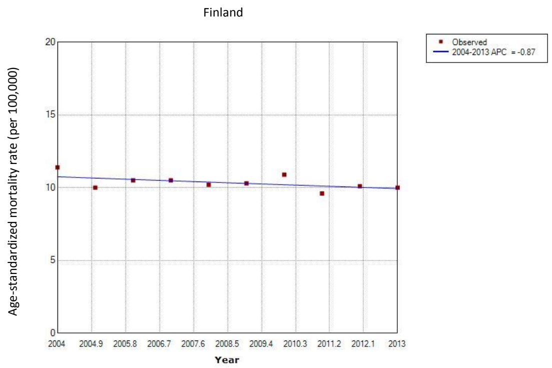
Finland
Observed
2004-2013 APC = -0.87
Age-standardized mortality rate (per 100,000)
Year

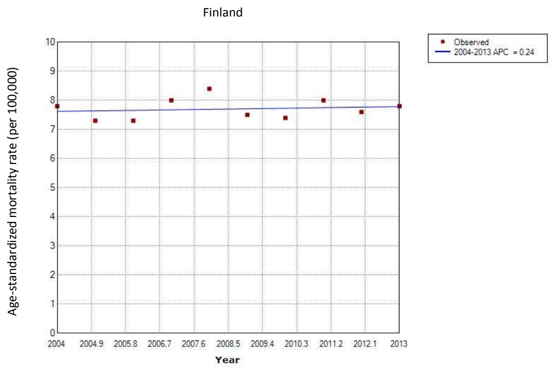
Finland
Observed
2004-2013 APC = 0.24
Age-standardized mortality rate (per 100,000)
Year

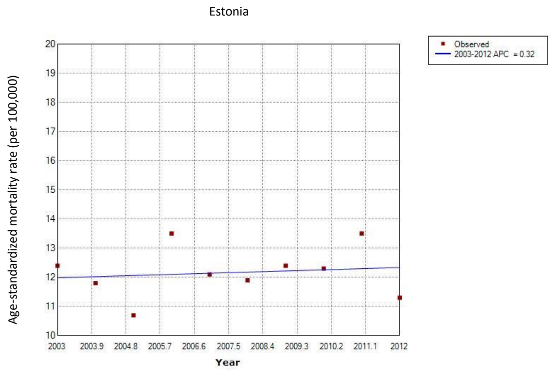
Estonia
Observed
2003-2012 APC = 0.32
Age-standardized mortality rate (per 100,000)
Year

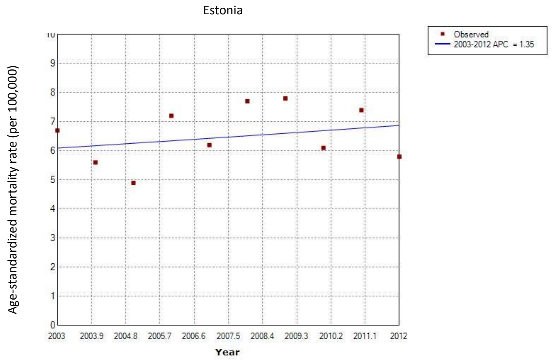
Estonia
Observed
2003-2012 APC = 1.35
Age-standardized mortality rate (per 100,000)
Year

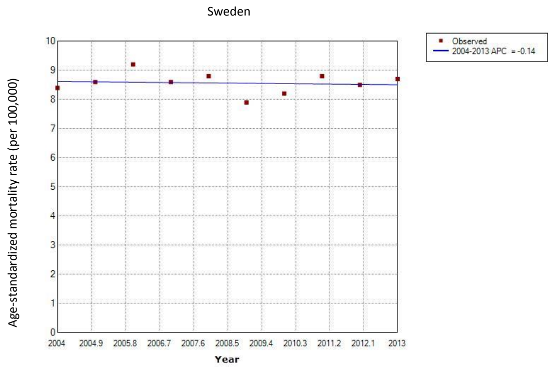
Sweden
Observed
2004-2013 APC = -0.14
Age-standardized mortality rate (per 100,000)
Year

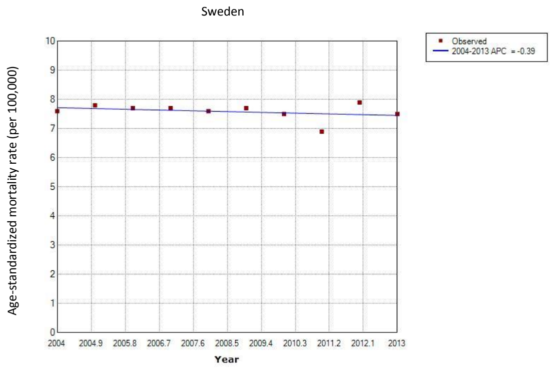
Sweden
Observed
2004-2013 APC = -0.39
Age-standardized mortality rate (per 100,000)
Year

Ireland

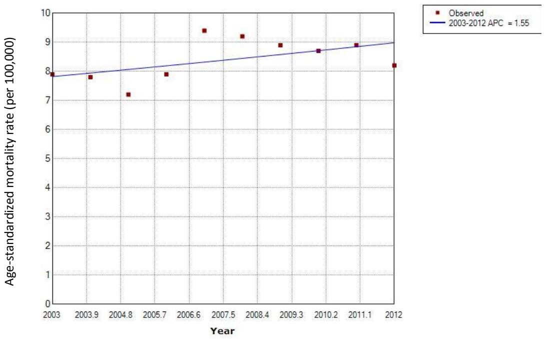

Ireland

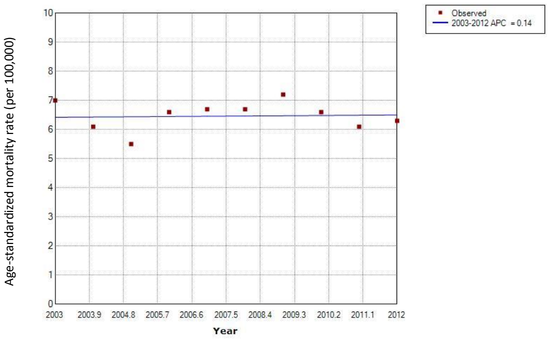

Denmark

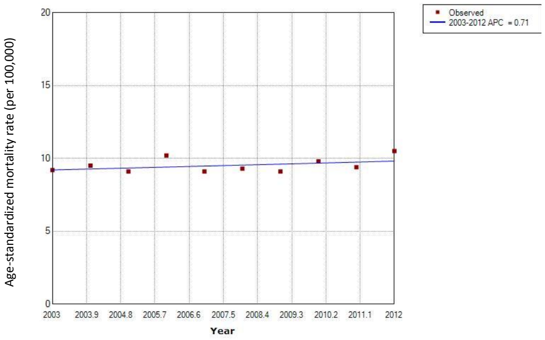

Denmark

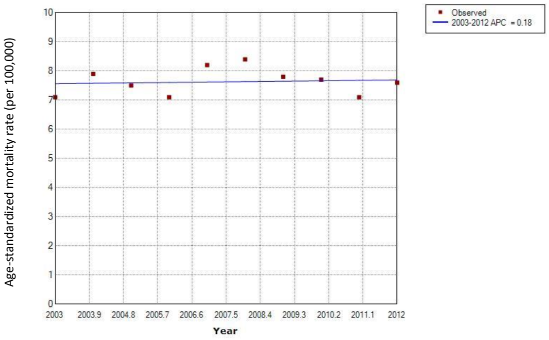

5) Western Europe

Netherlands

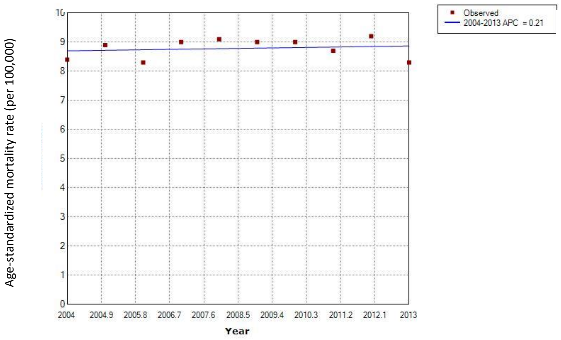

Netherlands

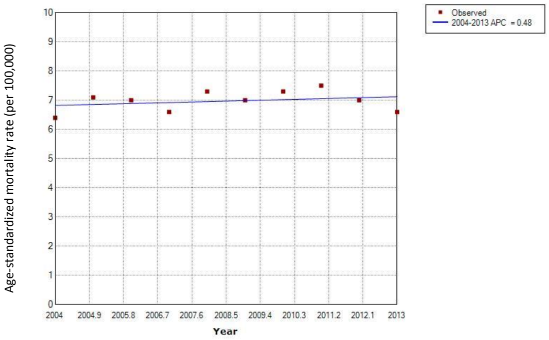

Switzerland

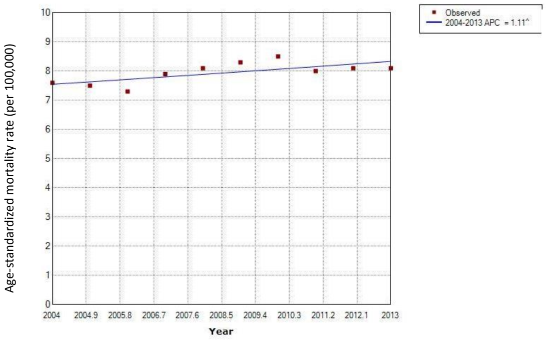

Switzerland

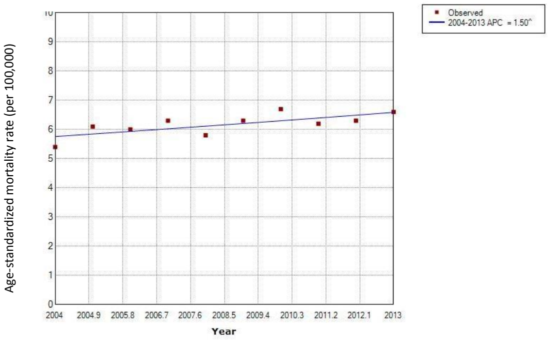

France

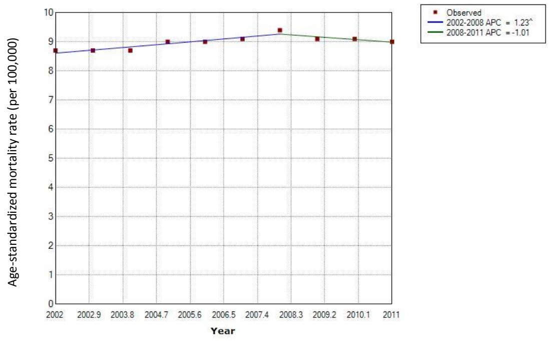

France

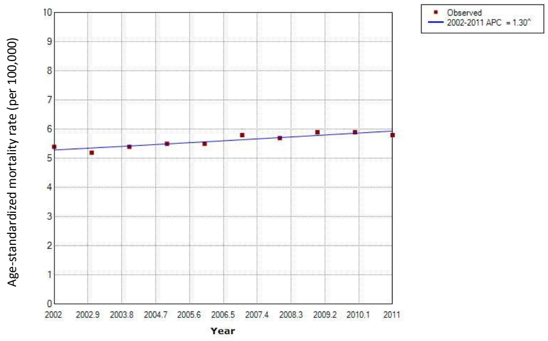

Austria

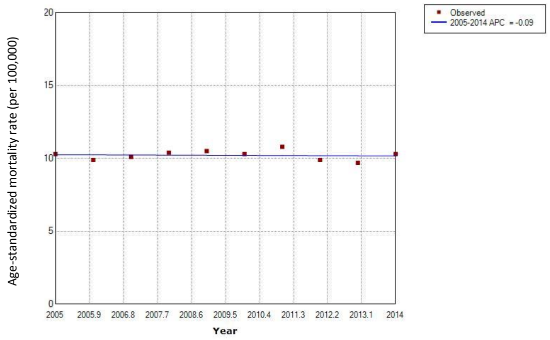

Austria

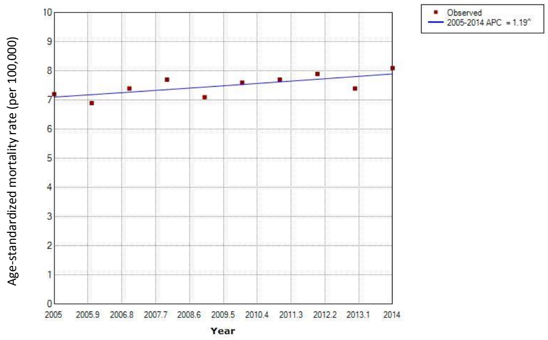

Germany

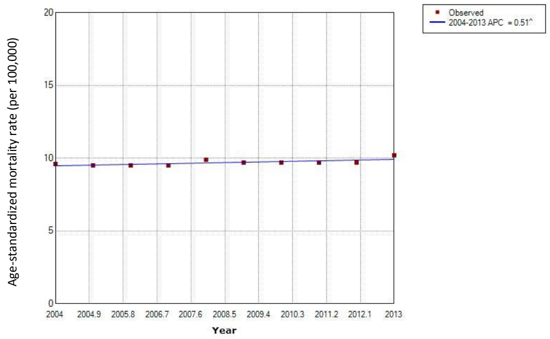

Germany

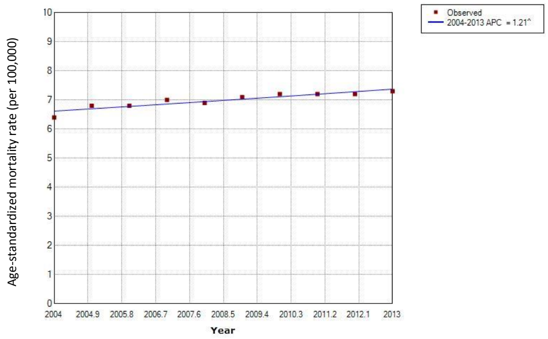

Belgium

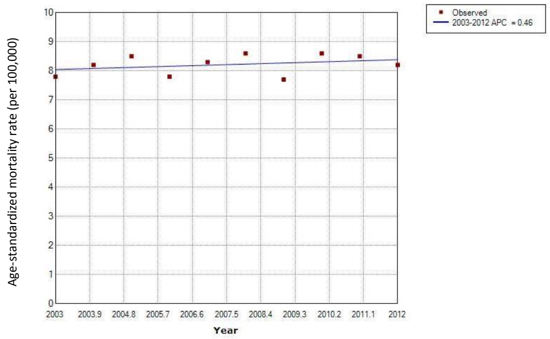

Belgium

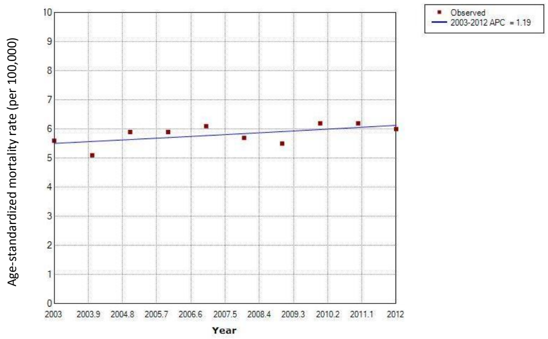

6) Southern Europe

Slovenia

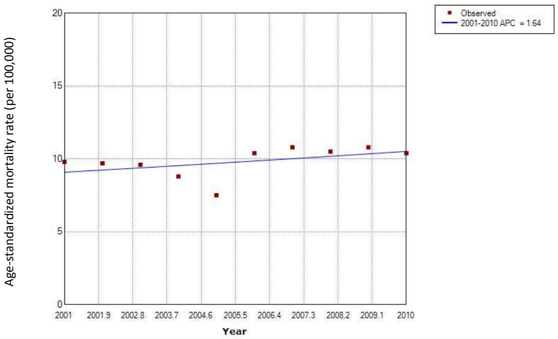

Slovenia

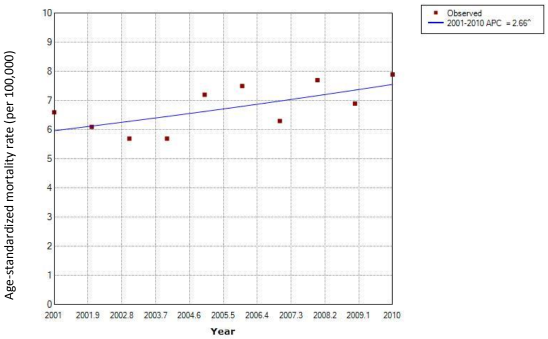

Spain

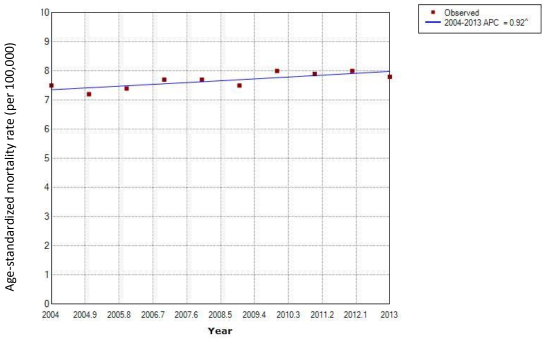

Spain

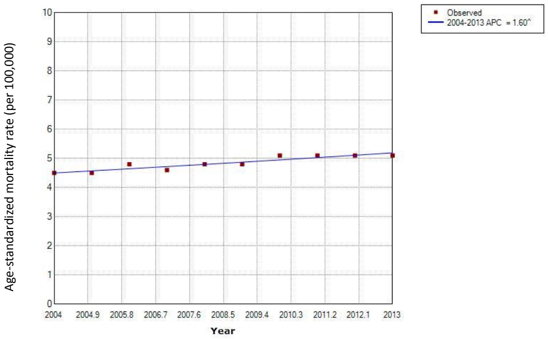

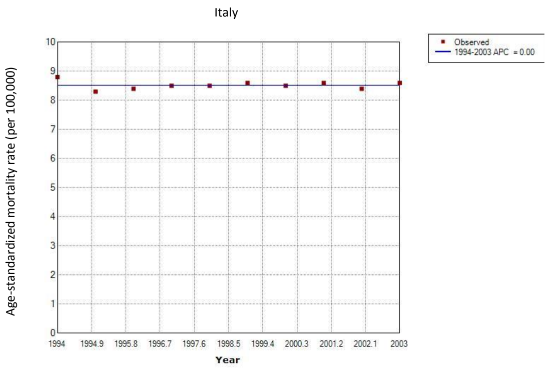

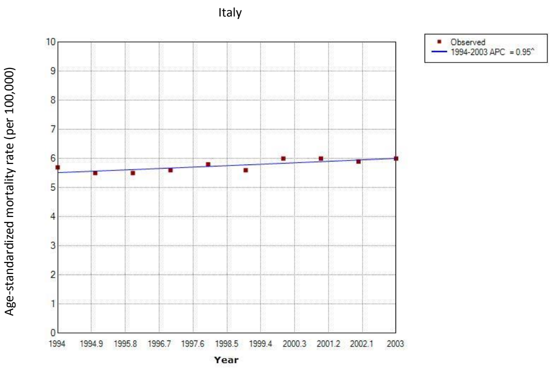

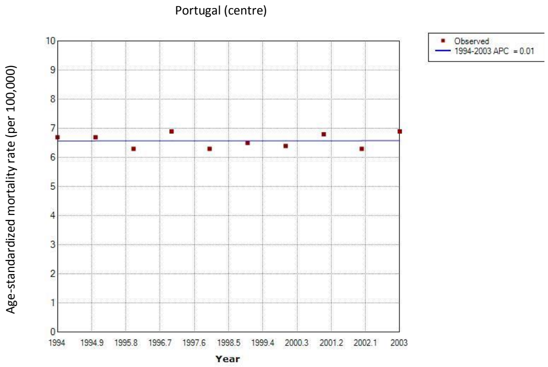

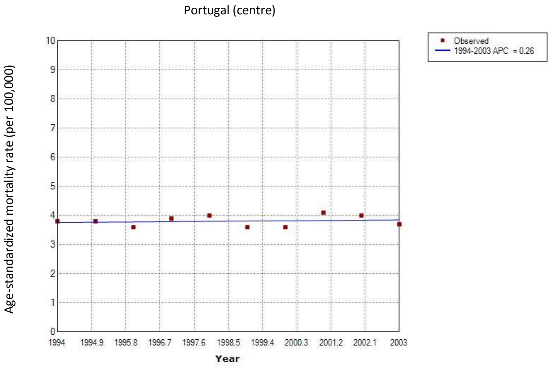

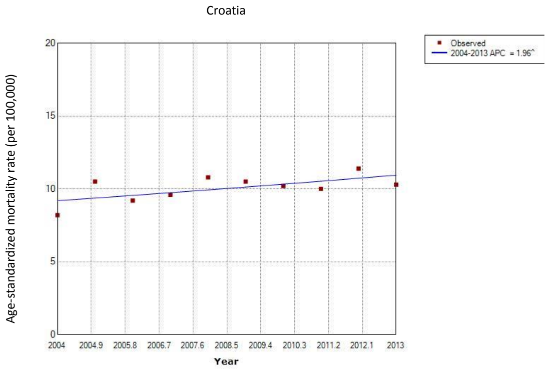

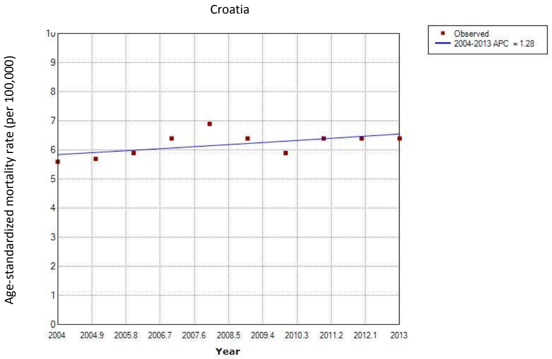

7) Eastern Europe

Russia

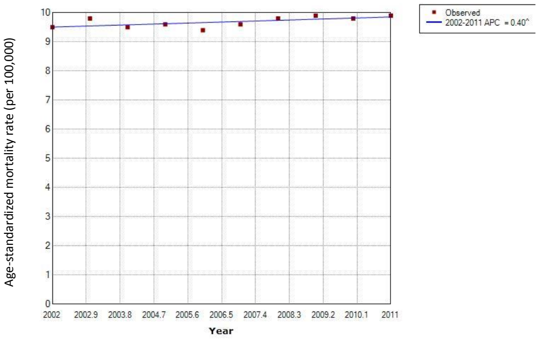

Russia

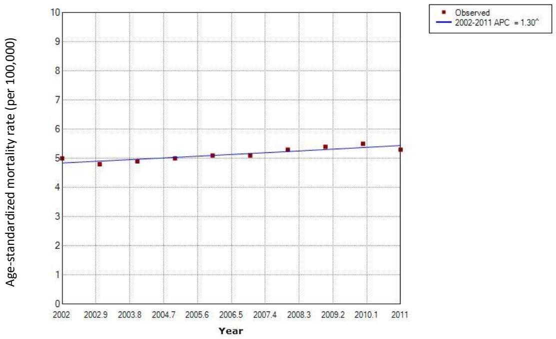

Poland

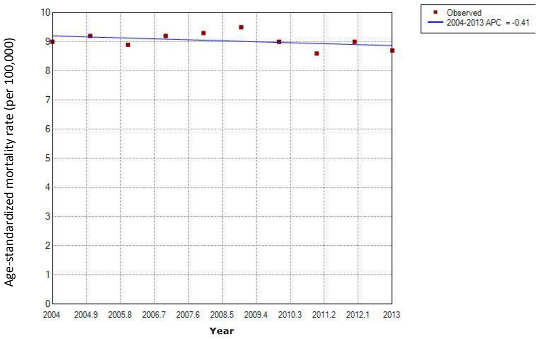

Poland

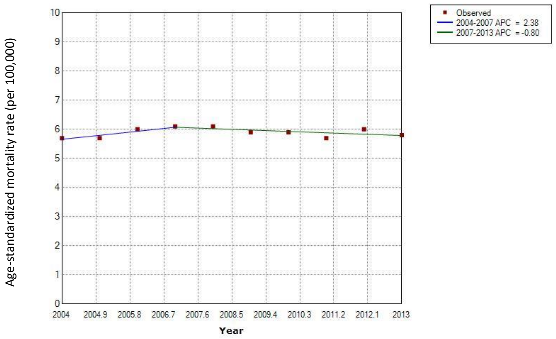

Slovakia

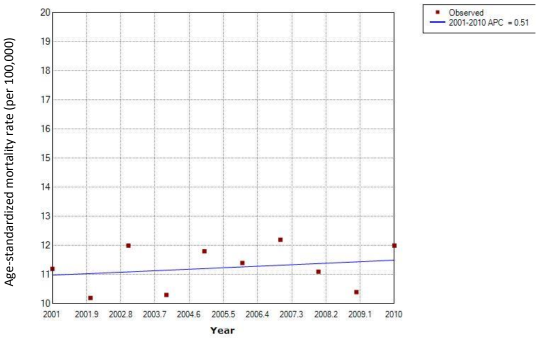

Slovakia

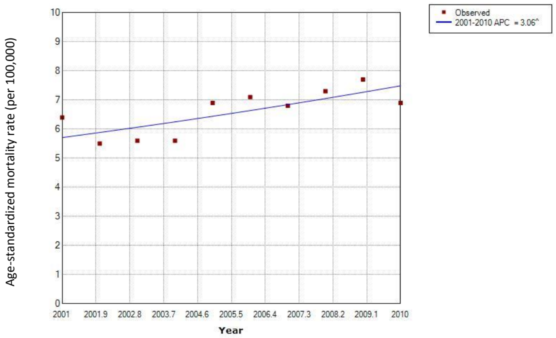

Czech Republic

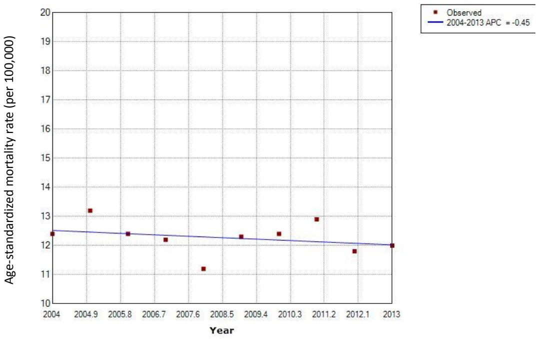

Czech Republic

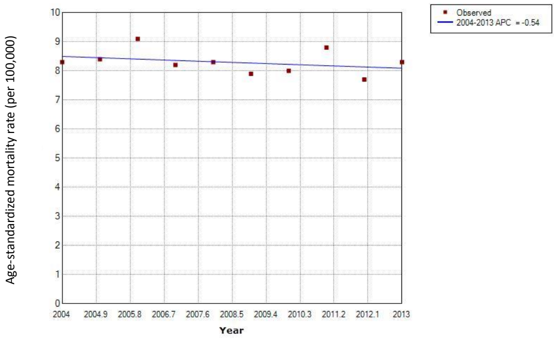

Supplement: Supplementary file 1 — Supplementary Figures [file 41598_2017_2997_MOESM1_ESM.pdf]
